# A Multifunctional Polar Amino Acid for Mixed Tin‐Lead Perovskites and All‐Perovskite Tandems

**DOI:** 10.1002/advs.202510740

**Published:** 2025-11-18

**Authors:** Jin Zhou, Hongsen Cui, Chen Wang, Dexin Pu, Lishuai Huang, Shun Zhou, Guang Li, Qingxian Lin, Shining Zhang, Weiqing Chen, Guojia Fang, Weijun Ke, Weiwei Meng

**Affiliations:** ^1^ College of Physics, Hebei Advanced Thin Films Laboratory Hebei Normal University Shijiazhuang 050024 China; ^2^ Key Laboratory of Artificial Micro‐ and Nano‐structures of Ministry of Education of China School of Physics and Technology Wuhan University Wuhan 430072 China; ^3^ College of Engineering Huazhong Agricultural University Wuhan 430070 China

**Keywords:** all‐perovskite tandems, electrostatic surface potential, polar amino acid, Sn‐Pb perovskites

## Abstract

Over the past years, the performance of all‐perovskite tandem solar cells has skyrocketed. However, mixed tin‐lead (Sn‐Pb) perovskites, which are pivotal in tandem cells, face challenges such as inherent Sn^2+^ oxidation and p‐type self‐doping. In this study, A molecular engineering strategy is introduced to address these issues by identifying key properties for effective additive designing: a high highest occupied molecular orbital level, a high boiling point, a large dipole moment, and a large electrostatic surface potential. Guided by these principles, the polar amino acid asparagine hydrochloride (AsnCl) is selected as a multifunctional additive. AsnCl, with its distinctive properties, effectively enhances the orientation and crystalline quality of perovskite films, suppresses harmful Sn^4+^ and PbI_2_ residues, realizes larger grains, and significantly extends carrier lifetimes while reducing non‐radiative recombination. As a result, the best‐performing single‐junction mixed Sn‐Pb perovskite solar cell achieves a power conversion efficiency (PCE) of 22.54% with significantly enhanced operational and storage stability. Furthermore, the two‐terminal all‐perovskite tandem solar cells based on AsnCl‐treated Sn‐Pb perovskites show high PCEs, and the highest steady‐state PCE is up to 28.24%. This work highlights the potential of additive molecular engineering strategies and their systematic selection principles in developing high‐performance perovskite tandem solar cells.

## Graphical abstract

AsnCl improves film orientation and crystallinity, suppresses Sn^4+^ and PbI_2_ defects, and prolongs carrier lifetimes, enabling a 22.54% efficiency and enhanced stability in Sn–Pb perovskite solar cells. The AsnCl‐treated tandem device achieves a steady‐state PCE of 28.24%, underscoring the effectiveness of additive molecular engineering for high‐performance PSCs.

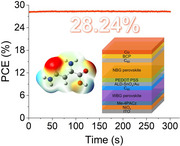

## 1 Introduction

Photovoltaics, as a clean and sustainable energy technology, has the potential to change the structure of global electricity production. Although crystalline silicon has dominated the photovoltaic field for decades, novel high‐performance semiconductors such as perovskites have emerged as promising candidates for the next generation of photovoltaic devices. Organic–inorganic metal halide perovskite solar cells (PSCs) have garnered considerable attention in recent years due to their high power conversion efficiencies (PCEs), cost‐effective fabrication, simple manufacturing process, and tunable bandgap across a broad range.^[^ ^1^ ^]^ Tremendous efforts devoted to this field have led to single‐junction perovskite solar cells achieving PCEs exceeding 26%.^[^ ^2^, ^3^, ^4^ ^]^ However, with their PCEs approaching the Shockley–Queisser (S‐Q) limit, further improvements in performance may become more challenging. To surpass the S‐Q limit of single‐junction cells, multi‐junction tandem configurations, such as crystalline silicon‐perovskite tandems and all‐perovskite tandems, were proposed, and they have demonstrated impressive potential.^[^ ^5^, ^6^, ^7^ ^]^ Notably, monolithic two‐terminal all‐perovskite tandem solar cells have made rapid progress in recent years.^[^ ^8^, ^9^, ^10^, ^11^, ^12^ ^]^ These tandems typically consist of a wide‐bandgap (WBG) lead‐halide perovskite subcell and a narrow‐bandgap (NBG) mixed tin‐lead (Sn‐Pb) perovskite subcell.^[^ ^13^, ^14^ ^]^ The NBG (≈1.25 eV) mixed Sn‐Pb perovskite solar cells are essential for high‐performance all‐perovskite tandems, as they allow more efficient utilization of near‐infrared solar irradiation, which normally cannot excite electron–hole pairs in WBG PSCs. Thus, various methods have been proposed to improve the performance of mixed Sn‐Pb PSCs. For example, Zhao et al. developed a bulk‐passivation approach based on chlorine incorporation.^[^ ^15^ ^]^ It was found that chlorine can enlarge grains and mitigate the electronic disorder in mixed Sn‐Pb perovskites, reducing trap‐assisted recombination and improving both PCEs and stability.^[^ ^15^ ^]^ Tong et al. improved carrier lifetimes and diffusion lengths by incorporating guanidinium thiocyanate into mixed Sn‐Pb perovskites, which resulted in higher photovoltaic parameters due to reduced defect density.^[^ ^16^ ^]^ In addition to bulk doping, interface treatments have been explored for mixed Sn‐Pb PSCs. Kapil et al. demonstrated that ethylenediamine can change the surfaces of mixed Sn‐Pb perovskite films from p‐type to n‐type, facilitating a favourable interface band bending that benefits carrier transport and reduces open‐circuit voltage (*V*_OC_) loss.^[^ ^17^ ^]^ Furthermore, other strategies, such as constructing low‐dimensional structures, have also been raised to improve the photovoltaic parameters and stability of mixed Sn‐Pb PSCs.^[^ ^18^ ^]^

The incorporation of Sn narrows the bandgap of perovskites and enhances their ability to harness solar infrared radiation. However, it also introduces challenges for the PCE and stability of PSCs due to the oxidation‐prone nature of Sn^2+^.^[^ ^19^ ^]^ The easy conversion from Sn^2+^ to Sn^4+^ critically constrains the photovoltaic properties of Sn‐Pb perovskites and hinders the further development of all‐perovskite solar cells.^[^ ^20^ ^]^ Sn vacancy defects, which result from the oxidation of Sn^2+^, can aggravate carrier recombination, hindering carrier transport and extraction, thereby reducing the photovoltaic performance of perovskite devices.^[^ ^21^, ^22^ ^]^ To address the challenge of Sn^2+^ oxidation in Sn‐containing perovskites, several methods have been proposed, including tin supplementation. For instance, Lin et al. reported that metallic tin powder can transform Sn^4+^ back to Sn^2+^, reducing Sn vacancies in mixed Sn‐Pb perovskites.^[^ ^23^ ^]^ Tin fluoride (SnF_2_), an additive that supplies additional Sn^2+^, has also been studied in Sn‐containing PSCs.^[^ ^24^, ^25^, ^26^ ^]^ Defect passivation strategies may further mitigate the defects caused by Sn^2+^ oxidation. Li et al. demonstrated that thiosemicarbazide strongly interacts with charged defects, increases the formation energy of Sn vacancy defects, and improves the performance of PSCs.^[^ ^22^ ^]^ Although substantial efforts have been devoted to reducing Sn^2+^ oxidation, more effective and multifunctional anti‐oxidation molecules remain needed, as Sn^2+^ oxidation continues to limit device performance. Furthermore, the mechanisms by which dopant molecules function in perovskites remain poorly understood, and the principles for additive selection are still unclear, posing significant challenges for further improving the performance of all‐perovskite tandem solar cells.

Herein, we establish an additive molecular selection principle to address the challenges in mixed Sn‐Pb perovskites by identifying key properties for effective additives: a high highest occupied molecular orbital (HOMO) level, a high boiling point, a large dipole moment, and a significant electrostatic surface potential. Guided by this framework, we selected the polar amino acid asparagine hydrochloride (AsnCl), whose ─COOH and ─NH_2_ moieties endow it with the desired characteristics. These properties effectively prevent Sn^2+^ oxidation throughout the preparation of precursors and film fabrication processes, thus reducing non‐radiative recombination and slowing the degradation of perovskites. Profiting from our strategy, the carrier lifetimes are greatly extended, and the photovoltaic parameters of PSCs are notably improved. The best‐performing mixed Sn‐Pb PSC achieved a PCE of 22.54% under a reverse current–voltage (*J*–*V*) scan, consistent with a steady‐state PCE of 22.54%, along with greatly improved storage and operational stability. We further incorporated AsnCl‐modified mixed Sn‐Pb perovskites into all‐perovskite tandem devices and achieved a steady‐state PCE of 28.24%, paving the way for highly efficient tandem solar cells.

## 2 Results and Discussion

The introduction of Sn^2+^ is an effective method for narrowing the bandgap of perovskites, which is crucial for fabricating efficient bottom subcells in all‐perovskite tandem solar cells. However, Sn^2+^ is intrinsically more prone to oxidation than Pb^2+^ due to Sn's higher atomic energy levels, lower electronegativity, and smaller splitting between *s* and *p* orbitals.^[^ ^21^ ^]^ The PCE and stability of mixed Sn‐Pb PSCs are constrained by the high density of Sn vacancies, which arise from the conversion of Sn^2+^ to Sn^4+^ in the surfaces and bulk of mixed Sn‐Pb perovskites. This oxidation leads to heavy p‐type self‐doping, which impedes carrier transport and extraction.^[^ ^27^, ^28^, ^29^ ^]^

To address the challenges outlined above, we screened potential functional molecules and selected a multifunctional Asn molecule. First, the ─COOH group can coordinate with Sn^2+^ and lift the density of localized electrons around Sn^2+^.^[^ ^30^ ^]^ Therefore, the coordination between Sn^2+^ and Asn is able to boost Sn^2+^’s antioxidative stability, which is a key factor for efficient and stable Sn‐Pb perovskite photovoltaic devices. Second, the ─NH_2_ group can act as an efficient grain surface‐passivator, interacting with the accepter‐like defects.^[^ ^31^ ^]^ Effective grain surface passivation is a vital method for improving the PCE and stability of mixed Sn‐Pb PSCs. Third, ─COOH and ─NH_2_ groups may have hydrogen‐bond interactions with I, which may lower the crystallization rate of perovskites and improve the crystalline quality.^[^ ^32^ ^]^

**Figure 1:**
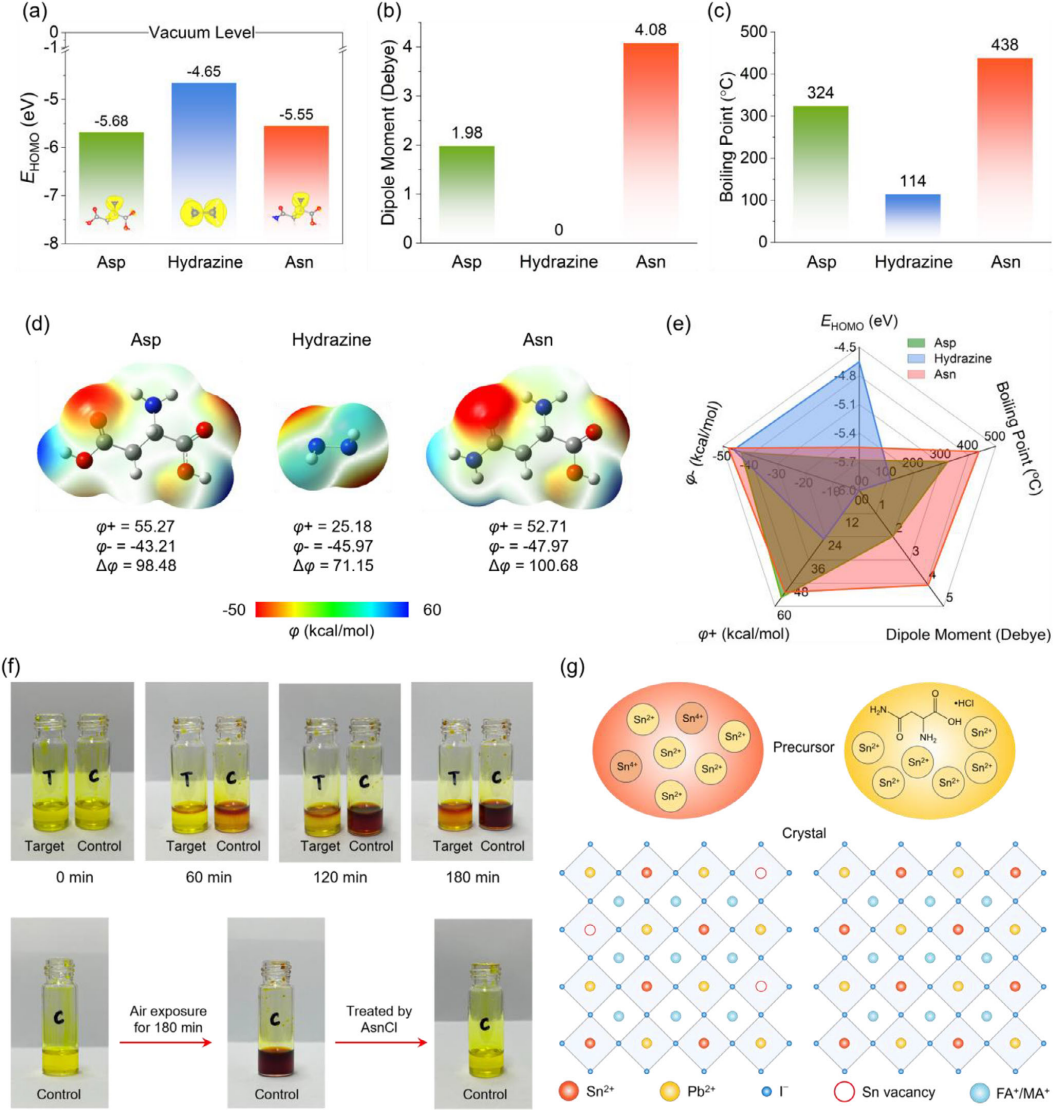
The theoretical analysis of molecules and the anti‐oxidation effects. a) The HOMO of Asn, Asp and hydrazine. b) The dipole moments of Asn, Asp, and hydrazine. c) The boiling points of Asn, Asp, and hydrazine. d) The distribution of electrostatic surface potential for Asn, Asp, and hydrazine. e) The radar plot for the representing molecular properties of Asn, Asp, and hydrazine. f) Photographs of precursor solutions without and with AsnCl addition exposed to ambient air. g) Schematic of depressing Sn^2+^ oxidation and decreasing Sn vacancy defects by AsnCl.

The ─COOH and ─NH_2_ groups are generally effective at enhancing perovskite film quality, leading to improved device performance.^[^ ^33^, ^34^ ^]^ In our previous work, we demonstrated that aspartate (Asp) can facilitate the preparation of high‐efficiency mixed Sn‐Pb PSCs and all‐perovskite tandem cells.^[^ ^33^ ^]^ However, due to the symmetry of the two terminal ─COOH functional groups in the architecture, the Asp exhibits a small dipole moment, which may limit its effective interaction with perovskite precursors. To improve the additive‐precursor interaction, we utilized the more electron‐donating ─NH_2_ group to replace one ─COOH group, which disrupts the symmetry of Asp, and results in the formation of the Asn molecule. This replacement brings a distinct change to the molecule's properties, enhancing its reducibility, as verified by density functional theory (DFT) analysis. As shown in **Figure** **1**a, the HOMO level of Asn is higher than that of Asp, indicating both a greater reducing ability and enhanced electron‐donating capability of Asn.^[^ ^35^ ^]^ Given that the ─NH_2_ group is significant for the reducibility of molecules, hydrazine, a molecule only composed of two ─NH_2_ groups, and its derivates are commonly recognized as typical additives with strong reducibility in Sn‐based PSCs.^[^ ^35^, ^36^, ^37^, ^38^ ^]^ As shown in Figure 1a, the HOMO of hydrazine is higher than those of Asn and Asp, indicating its distinct reducibility. However, due to its highly symmetrical molecular architecture, hydrazine maintains a dipole moment of 0 Debye (Figure 1b), which is unfavorable for the interaction between additives and perovskites. Moreover, the high biotoxicity hinders the application of hydrazine and its derivates. By contrast, in terms of molecular structure, Asn benefits from greater asymmetry than Asp and hydrazine, resulting in a larger dipole moment (Figure 1b). The increased dipole moment can facilitate its interactions with perovskites and consequently improve the defect passivation effect.^[^ ^39^ ^]^ Notably, Asn has a higher boiling temperature than those of Asp and hydrazine, allowing for better manipulation and improvement of the crystallization of mixed Sn‐Pb perovskites (Figure 1c).^[^ ^40^, ^41^ ^]^ To make the electronic properties of the molecules visualized, the electrostatic surface potential (ESP) is depicted in Figure 1d. The distribution of ESP further highlights the molecular structure's influences on the molecules’ defect coordination and passivation capabilities.^[^ ^42^ ^]^ For a dopant to function as an effective passivator, it should fulfill two primary criteria: it possesses functional groups for passivation, and it exhibits a strong anchoring affinity to the perovskite grain surfaces.^[^ ^31^ ^]^ It is worth noting that the intensity of passivator adsorption significantly depends on the distribution of ESP and dipole moments. The binding energy between passivators and defects is positively correlated with the potential difference between electron‐rich and electron‐poor ends of molecules.^[^ ^31^, ^43^ ^]^ Thus, this potential difference can enhance the adsorption capability of passivators. As shown in Figure 1d, the most prominent characteristic in the ESP of hydrazine is its perfect symmetry, which is determined by its structure and is consistent with its dipole moment being as small as 0 Debye. However, such a small dipole moment would restrict hydrazine to construct strong interactions, such as hydrogen bonds, and coordinate with charged defects in perovskite films.^[^ ^44^ ^]^ Since high‐temperature annealing is typically necessary for perovskite film formation, passivators with small dipole moments like hydrazine are prone to desorb from perovskites and lead to the formation of defects. Therefore, although hydrazine features strong reducibility and two most typical passivating groups, the small molecular polarity limits its defect passivation and manipulation of crystallization. In other words, it is challenging for hydrazine to act as a multifunctional additive that can simultaneously protect Sn^2+^ from oxidation and achieve efficient defect passivation in mixed Sn‐Pb perovskites. By contrast, in the ESP map, Asn exhibits the largest potential difference between the electron‐rich and electron‐poor ends compared to Asp and hydrazine, which aligns with its largest dipole moment, resulting in the most stable surface adsorption and the most efficient defect passivation.^[^ ^31^ ^]^ We then compared five representative properties of molecules beneficial for improving the performance of PSCs, as illustrated in the radar plots of Figure 1e. Owing to the reduced molecular symmetry, Asn achieved the best trade‐off among the HOMO level, the dipole moment, the ESP, and the boiling point, suggesting its suitability for advancing perovskite functionality.

Since Asn has been selected as a multifunctional additive according to the theoretical analysis, we further conducted the protonation of the ─NH_2_ groups using hydrochloric acid (HCl) to form the corresponding amino acid salt. This step was partly motivated by the reported positive effects of chloride ions (Cl^−^) on grain enlargement in mixed Sn‐Pb perovskites.^[^ ^15^ ^]^ Moreover, recent studies have shown that HCl‐protonated amino acids represent a promising class of passivators for PSCs owing to their multifunctional defect‐passivation capabilities. The HCl protonation introduces additional –NH_3_^+^ and Cl^−^ functionalities that enable synergistic interactions with both anionic and cationic defects, thereby suppressing ion migration and mitigating detrimental defect states.^[^ ^45^ ^]^ Typically, Sn^2+^ oxidation begins in perovskite precursor solutions, resulting in shortened carrier lifetimes and diffusion lengths, ultimately impairing carrier transport and extraction. This transformation of Sn^2+^ to Sn^4+^ diminishes the photovoltaic performance of both mixed Sn‐Pb PSCs and their tandems. To validate AsnCl's effect in mitigating Sn^2+^ oxidation in perovskite precursor solutions, we photographed the precursor solutions prepared by dissolving formamidinium iodide (FAI), methylammonium iodide (MAI), tin iodide (SnI_2_), lead iodide (PbI_2_), SnF_2_, and lead thiocyanate (Pb(SCN)_2_) in a mixed solvent of dimethylformamide (DMF) and dimethylsulfoxide (DMSO). The control solution and the target solution (that is, the precursor solution with 3 mol% AsnCl molecules) were exposed to ambient air (≈26 °C, 50% RH). As shown in Figure 1f, the control solution's color changed from yellow to dark red after 180 min, signaling the oxidation of Sn^2+^ to Sn^4+^. By contrast, the AsnCl‐modified solution exhibited only a slight color change under the same conditions, demonstrating that AsnCl effectively prevents Sn^2+^ oxidation. Notably, the aged control solution also reverted from red to yellow after the addition of AsnCl, suggesting that AsnCl molecules can restore Sn^4+^ to Sn^2+^. By preventing Sn^2+^ oxidation, AsnCl reduced the presence of Sn^4+^, contributing to the formation of mixed Sn‐Pb perovskites with low Sn vacancy defects (Figure 1g).

**Figure 2:**
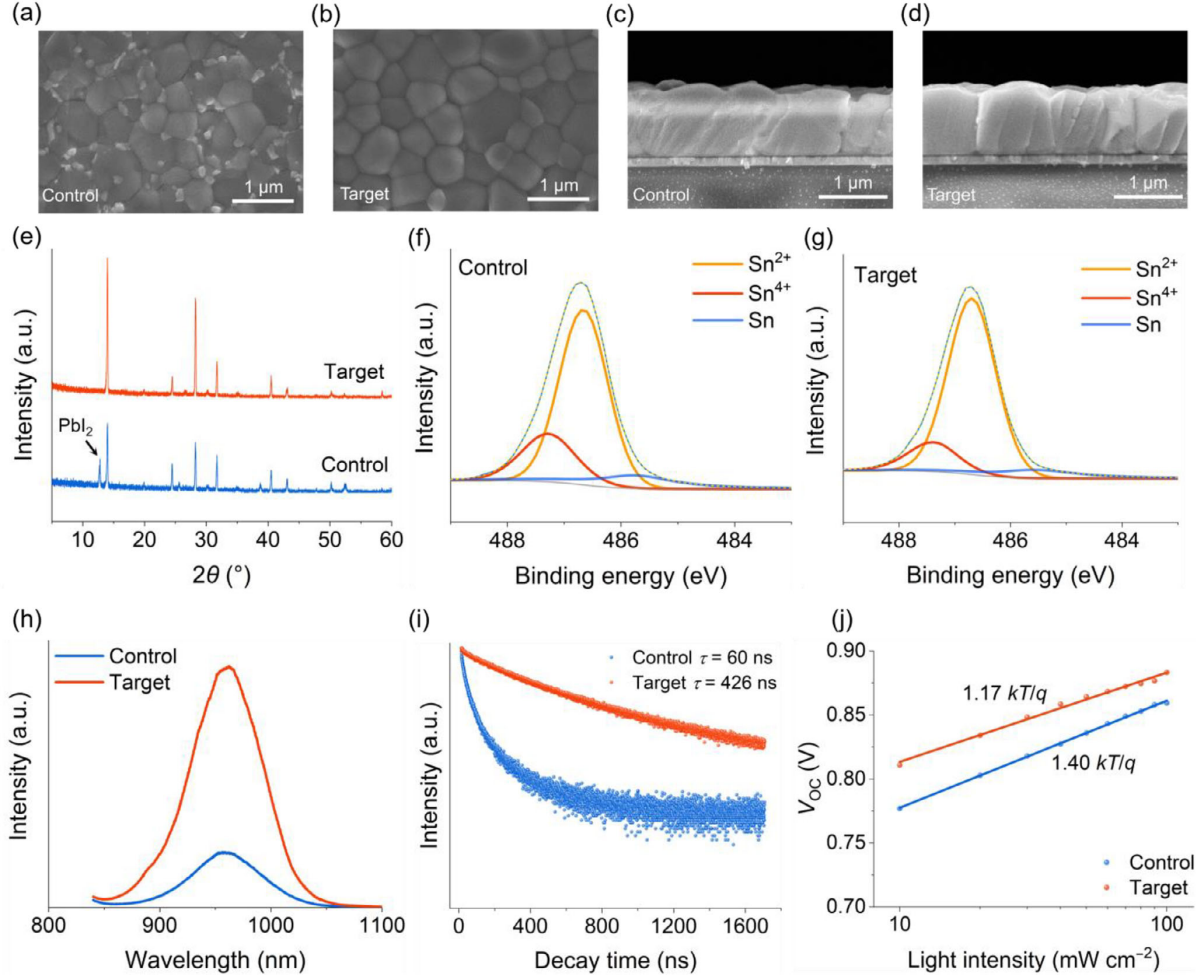
The experimental characterizations of perovskites and single‐junction devices. Top‐view SEM images of a) control and b) AsnCl‐treated perovskites. Cross‐sectional SEM images of c) control and d) AsnCl‐treated perovskites. e) XRD patterns of mixed Sn‐Pb perovskites without and with AsnCl addition. XPS spectra of Sn‐Pb perovskites without f) and with g) AsnCl. PL spectra h) and i) TRPL decay curves of control and AsnCl‐treated perovskite films. j) Relationship between *V*_OC_ and varying light intensity for control and AsnCl‐treated devices.

To investigate the effect of AsnCl dopants on the crystallization of perovskites, we prepared mixed Sn‐Pb perovskite films with the composition FA_0.7_MA_0.3_Sn_0.5_Pb_0.5_I_3_ using both control and AsnCl‐modified precursor solutions. For the AsnCl‐modified solutions, 1.5 mol% (relative to A‐cite cations) of AsnCl was added. The changes in crystallization were observed through scanning electron microscopy (SEM), as shown in **Figure** **2**a–d. In the presence of AsnCl molecules, the surface of the mixed Sn‐Pb perovskite films appeared cleaner, with almost no visible unwanted non‐perovskite phases or components such as PbI_2_ (Figure 2a,b). Cross‐sectional SEM images further revealed that AsnCl dopants promote grain growth in the vertical direction (Figure 2c,d). The reduced number of longitudinal grain boundaries in AsnCl‐incorporated Sn‐Pb perovskite films is advantageous for carrier transport, as grain boundary defects can trap carriers and aggravate non‐radiative recombination losses.

To further investigate the effect of AsnCl on the perovskite structure, we conducted an X‐ray diffraction (XRD) analysis of mixed Sn‐Pb perovskites with varying concentrations of AsnCl. The XRD patterns exhibited no distinct peak at low angles (<10°), indicating that the mechanism by which AsnCl molecules function is likely unrelated to the formation of low‐dimensional perovskite phases (Figure 2e). For the perovskite with 1.5% AsnCl, the diffraction intensity of the (100) lattice plane was significantly higher than that of the control perovskites. We further calculated the diffraction intensity ratios of the (100) plane to the (111) and (210) planes, as depicted in Figure S1 (Supporting Information). The increased ratios of both (100)/(111) and (100)/(210) suggest that AsnCl improved the crystallinity of perovskites. The crystallographic properties of mixed Sn‐Pb perovskites were further explored via the pole figure measurements. As shown in Figure S2 (Supporting Information), the diffraction intensity corresponding to (100) facet orientation in the target film was distinctly enhanced, evidencing the improved grain growth orientation caused by the doping of AsnCl.^[^ ^46^ ^]^ The results of pole figure measurements are consistent with the XRD and SEM images in Figure 2. The facet orientation tailoring can be ascribed to AsnCl's high boiling temperature and strong adsorption capability, which is determined by its large dipole moment. Such strong adsorption helps AsnCl function as a grain growth manipulator in the high‐temperature film formation process. The enhanced crystallization orientation can boost the carrier transport in cells and lead to improved photovoltaic parameters. Additionally, the XRD patterns of the control perovskites without AsnCl showed a distinct PbI_2_ diffraction peak, indicating the presence of residual PbI_2_ in the final films. In contrast, no discernible PbI_2_ signal was observed in the AsnCl‐modified perovskites, which could mainly be attributed to the strong binding between AsnCl and PbI_2_. To further verify this effect, we conducted XRD measurements on mixed Sn‐Pb perovskite films with excess PbI_2_ (5%). As shown in Figure S3 (Supporting Information), the AsnCl‐modified film exhibited a reduced PbI_2_ diffraction peak compared to the control film, consistent with the observations in Figure 2e.^[^ ^47^ ^]^ Residual PbI_2_ on the surface of perovskite films can reduce the operational stability of devices and accelerate device degradation.^[^ ^48^ ^]^ Thus, it is reasonable to infer that the interaction between AsnCl and PbI_2_ positively influences the stability of mixed Sn‐Pb perovskites and their devices.

As demonstrated earlier, AsnCl effectively suppressed Sn^2+^ oxidation in precursor solutions. Given that Sn^2+^ oxidation can occur in both precursor solutions and perovskite films, we further investigated Sn^2+^ oxidation in fabricated perovskite films using X‐ray photoelectron spectroscopy (XPS) analysis. As shown in Figure 2f,g, the Sn^4+^ content was reduced due to the anti‐oxidation effect of AsnCl doping. Since non‐radiative recombination caused by defects is a major factor limiting the photovoltaic performance of PSCs, we also examined the effects of AsnCl on defect passivation and non‐radiative recombination in mixed Sn‐Pb perovskites. First, we studied the photophysical characteristics of the mixed Sn‐Pb perovskite films. Perovskite films were prepared on indium tin oxide (ITO) substrates, and steady‐state photoluminescence (PL) measurements were conducted to assess the defect passivation effects of AsnCl molecules. As shown in Figure 2h, the steady‐state PL intensity of AsnCl‐incorporated perovskites was noticeably higher than that of the control perovskites, suggesting effective defect passivation and a subsequent reduction in non‐radiative recombination losses due to AsnCl doping.^[^ ^49^, ^50^ ^]^ For high short‐circuit current density (*J*_SC_) in all‐perovskite tandems, the thickness of mixed Sn‐Pb perovskites must be increased to match the high photocurrent density of the front WBG subcell. As a result, long carrier lifetimes and diffusion lengths are essential for high‐performance NBG PSCs. Time‐resolved PL (TRPL) characterizations further confirmed the positive impact of AsnCl molecules on charge‐carrier dynamics in mixed Sn‐Pb perovskites. The carrier lifetime *τ* was calculated using the equation:

(1) $$\tau =\frac{A_{1}\tau_{1}^{2}+A_{2}\tau_{2}^{2}}{A_{1}\tau_{1}+A_{1}\tau_{2}}$$

where *τ*_1_ and *τ*_2_ are decay time constants, *A*_1_ and *A*_2_ are the corresponding amplitudes of decay. As shown in Figure 2i, the carrier lifetime of the AsnCl‐modified sample (426 ns) was significantly longer than that of the control sample (60 ns). The prolonged carrier lifetimes in AsnCl‐incorporated samples could enhance efficient carrier transport, enabling mixed Sn‐Pb perovskite absorbers to fully utilize photon‐generated carriers. In addition to long carrier lifetimes, achieving high *J*_SC_ in all‐perovskite tandems requires efficient absorption across the visible to near‐infrared range. However, the UV–vis absorption spectra showed no significant differences in absorption curves between AsnCl‐modified and control perovskite films (Figure S4, Supporting Information). Moreover, the bandgaps of the perovskites, determined by the Gaussian distribution of dEQE/dE, remained unchanged at ≈1.25 eV even with AsnCl incorporation (Figure S5, Supporting Information).

To study the carrier transport and recombination dynamics in perovskite films, we conducted dark *J*–*V* curves and electronic impedance spectroscopy (EIS) of the fabricated Sn‐Pb perovskite devices. For the AsnCl‐modified device, the reverse saturation current was obviously lower than that of the control device (Figure S6, Supporting Information), which can be attributed to the reduced background carrier density induced by AsnCl doping.^[^ ^51^, ^52^ ^]^ As shown in Figure S7 (Supporting Information), the recombination resistance significantly increased, indicating that carrier recombination was effectively suppressed due to the passivation effects of AsnCl molecules. We further assessed carrier recombination losses by investigating the dependence of open‐circuit voltage (*V*_OC_) on light intensity (*P*). The measured data were fitted to the following linear relationship:

(2) $$V_{\mathrm{OC}}\propto \frac{nkT}{q\ln P}$$

where *q* is the electron charge, *k* denotes the Boltzmann constant, and *T* represents the absolute temperature. As shown in Figure 2j, the fitted slope for the AsnCl‐modified sample was 1.17 *kT* *q* ^−1^, significantly smaller than that of the control sample (1.40 *kT* *q* ^−1^). A reduced slope indicates decreased trap‐induced carrier recombination under open‐circuit conditions, suggesting a lower trap density and inhibited defects‐assisted recombination as a result of AsnCl doping.^[^ ^53^ ^]^ Mott‐Schottky analysis was conducted to determine the built‐in potential (*V*_b_) of mixed Sn‐Pb PSCs, using the following equation:

(3) $$C^{2}=\frac{2\left(V_{b}-V\right)}{A^{2}e\varepsilon \varepsilon_{0}N}$$

where *A* denotes the surface area, *e* means the elementary charge, ε_0_ is the vacuum permittivity, ε represents the relative dielectric constant, *N* expresses the charge density, and *V* is the symbol of applied voltage in measurements. The *V*_b_ of the AsnCl‐modified device was 0.56 V, compared to 0.49 V for the control device (Figure S8, Supporting Information), indicating a stronger electric field that contributes to a higher *V*_OC_ in the devices.

We also analyzed the valence band maximum (VBM), the conduction band minimum (CBM), and the Fermi level of perovskites through ultraviolet photo‐electron spectroscopy measurements (Figure S9, Supporting Information). As depicted in Figure S10 (Supporting Information), the Fermi level in the AsnCl‐treated perovskites was closer to the VBM compared to the control sample, indicating a stronger n‐type character and reduced p‐type self‐doping due to AsnCl treatment.

**Figure 3:**
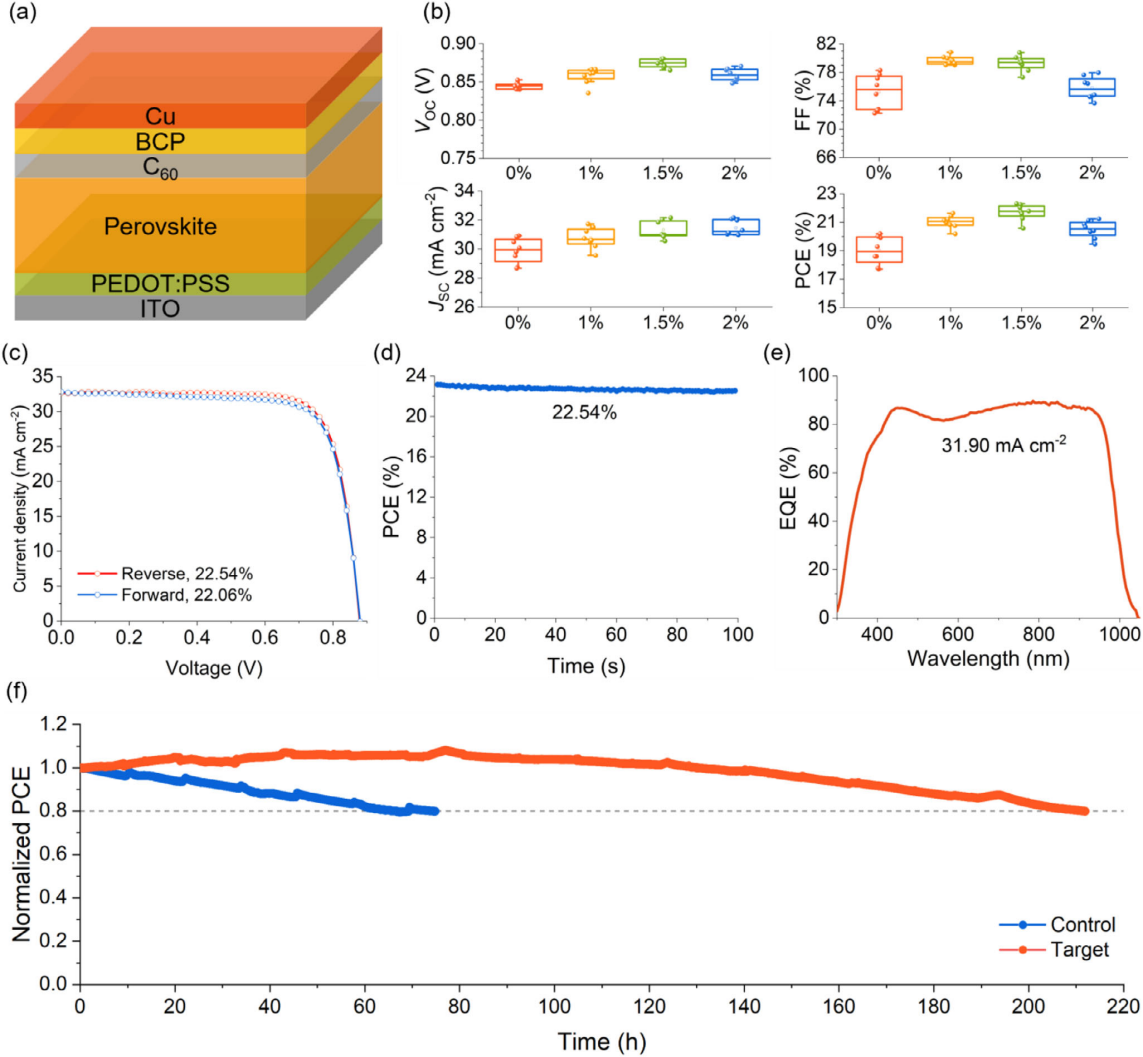
The structure and photovoltaic parameters of mixed Sn‐Pb perovskite cells. a) Schematic of the p‐i‐n configuration of mixed Sn‐Pb PSCs. b) Photovoltaic parameter statistics of mixed Sn‐Pb PSCs with varying AsnCl concentrations. c) *J*–*V* curves of the best‐performing single‐junction mixed Sn‐Pb PSC. d) Steady‐state PCE of the best‐performing mixed Sn‐Pb PSC. e) EQE spectrum of the AsnCl‐modified PSC. f) MPPT measurements of control and AsnCl‐treated devices.

After assessing the enhanced optoelectrical properties of perovskite films with AsnCl doping, we fabricated single‐junction mixed Sn‐Pb PSCs to evaluate the impact of AsnCl on device performance. The p‐i‐n structure of these PSCs, consisting of an ITO electrode, hole transport layer (PEDOT:PSS), perovskite absorber, electron transport layer (C_60_), and Cu electrode, is illustrated in **Figure** **3**a. To identify the optimal AsnCl doping concentration, we assessed the photovoltaic parameters, including *V*_OC_, *J*_SC_, fill factor (FF), and PCE, for mixed Sn‐Pb PSCs with various AsnCl concentrations, with the results presented in Figure 3b. The optimal AsnCl doping concentration was determined to be 1.5 mol% (relative to A‐site cations). As shown in Figure 3b, the AsnCl‐treated devices exhibited evidently higher *J*_SC_ than the control devices, attributed to reduced trap density and suppressed non‐radiative recombination, rather than enhanced light absorption, since both AsnCl‐treated and control perovskites displayed similar UV‐vis absorption spectra (Figure S4, Supporting Information). To evaluate the photovoltaic parameters of the AsnCl‐modified devices, we fabricated 16 devices and conducted statistical analysis (Figure S11, Supporting Information). The AsnCl‐treated devices exhibited an average *V*_OC_ of 0.87 ± 0.004 V, an average *J*_SC_ of 32.04 ± 0.26 mA cm^−2^, an average FF of 79.20 ± 0.64%, and an average PCE of 21.99 ± 0.27%. For comparison, we also fabricated 16 aspartate hydrochloride (AspCl)‐modified mixed Sn‐Pb PSCs and presented the statistics of their photovoltaic parameters in Figure S11 (Supporting Information). The AspCl‐modified devices showed an average *V*_OC_ of 0.85 ± 0.004 V, an average *J*_SC_ of 32.00 ± 0.29 mA cm^−2^, an average FF of 79.53 ± 0.53%, and an average PCE of 21.63 ± 0.30%. The performance of AsnCl‐based devices was superior to AspCl‐based devices, which agreed well with our theoretical molecular analysis. The best‐performing AsnCl‐treated PSC achieved a PCE of 22.54% (reverse *J*–*V* scan) with a *V*_OC_ of 0.88 V, a *J*_SC_ of 32.92 mA cm^−2^, and an FF of 78.04%. For the forward *J*–*V* scan, the PCE was 22.06%, with a *V*_OC_ of 0.88 V, a *J*_SC_ of 33.10 mA cm^−2^, and an FF of 75.85% (Figure 3c). The steady‐state PCE reached 22.54%, consistent with the *J*–*V* scan results (Figure 3d). Current density from *J*–*V* scans was further validated by EQE measurements (Figure 3e), with an integrated current density of 31.90 mA cm^−2^, closely matching the *J*–*V* scan results.

Beyond PCE improvements, the integration of AsnCl also enhanced both operational stability and storage stability. Long‐term stability tests were conducted by storing the mixed Sn‐Pb PSCs in an N_2_‐filled glovebox at room temperature. The AsnCl‐treated devices retained over 90% of their initial PCE after 2500 h, whereas the control sample's PCE declined to 88% after 1636 h (Figure S12, Supporting Information). This improvement indicates that AsnCl effectively retards the degradation of mixed Sn‐Pb Perovskites. Additionally, we performed maximum power point tracking (MPPT) tests to evaluate the operational stability of the devices. The unencapsulated control and AsnCl‐treated devices were aged under 1‐sun illumination in an N_2_‐filled glovebox at ≈55 °C. As shown in Figure 3f, the PCE of the control PSC decreased by 20% after 64 h of constant MPPT, while the AsnCl‐treated device maintained 80% of its initial PCE after 210 h. Non‐radiative recombination caused by Sn vacancy defects, the products of Sn^2+^ oxidation, is a primary inducement to the performance decay of mixed Sn‐Pb perovskite cells and their tandem cells. Therefore, the enhanced long‐term storage stability and operational stability of devices should be ascribed to the depressed Sn^2+^ oxidation and effective defect passivation supported by the doping of AsnCl.

**Figure 4:**
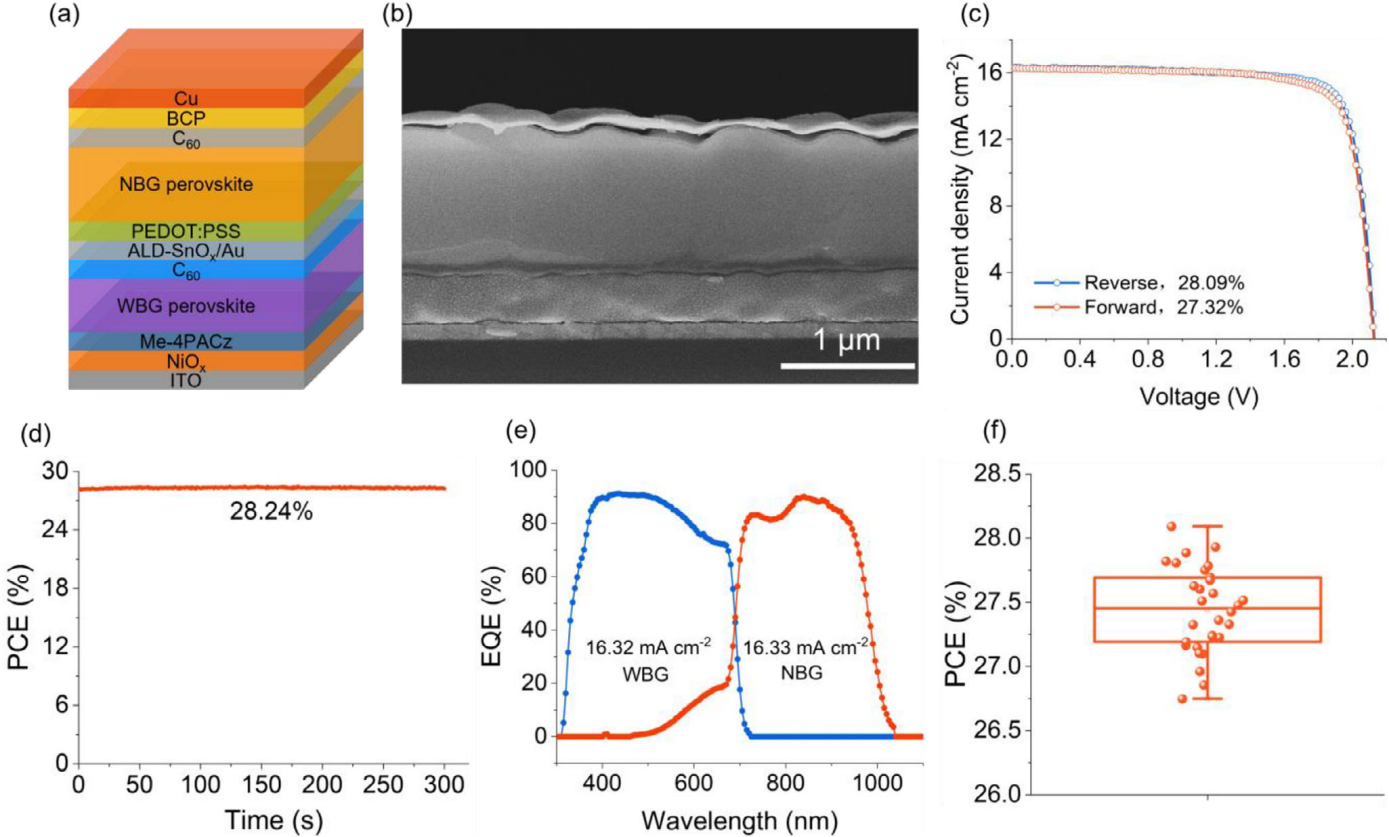
The structure and performance of all‐perovskite tandem cells. a) Schematic and b) cross‐sectional SEM image of all‐perovskite tandem cells. c) *J*–*V* curves, d) steady‐state PCE, and e) EQE spectra of the best‐performing all‐perovskite tandem cell. f) PCE statistics of AsnCl‐integrated all‐perovskite tandem cells.

As discussed, all‐perovskite tandem solar cells have emerged as strong candidates for commercial photovoltaic applications and are regarded as one of the most promising pathways in perovskite photovoltaics. High‐quality mixed Sn‐Pb perovskites play a crucial role in fabricating efficient all‐perovskite tandem solar cells. Building on this, we integrated the optimized Sn‐Pb perovskite subcells into monolithic all‐perovskite tandem cells. These tandem solar cells featured a structure of ITO/NiO_x_/Me‐4PACA/WBG perovskite/C_60_/ALD‐SnO_x_/Au/PEDOT:PSS/NBG perovskite/C_60_/BCP/Cu, with the schematic diagram shown in **Figure** **4**a. Figure 4b presents a cross‐sectional SEM image of a representative AsnCl‐incorporated all‐perovskite tandem. Figure 4c shows the *J*–*V* curves for our best‐performing all‐perovskite tandem solar cell, which achieved a PCE of 28.09% under a reverse *J*–*V* scan, with a *V*_OC_ of 2.13 V, a *J*_SC_ of 16.35 mA cm^−2^, and an FF of 80.74%. The steady‐state PCE was 28.24%, as depicted in Figure 4d. To confirm the accuracy of *J*_SC_ for the best‐performing all‐perovskite tandem, we also performed EQE measurements. The NBG perovskite subcell showed an integrated *J*_SC_ of 16.33 mA cm^−2^, which aligned with the WBG subcell's *J*_SC_ of 16.32 mA cm^−2^. This well‐balanced current density between the NBG and WBG subcells, supported by reduced defect density and enhanced carrier transport through AsnCl doping, was a key contributor to the high PCEs of the all‐perovskite tandems. To ensure the reproducibility of these high photovoltaic parameters, we fabricated 30 individual all‐perovskite tandem devices (with an aperture area of 0.070225 cm^2^). The histograms of their PCE, *V*_OC_, *J*_SC_, and FF are provided in Figure 4f and Figure S13 (Supporting Information). These tandems demonstrated an average PCE of 27.44 ± 0.33%, an average *V*_OC_ of 2.12 ± 0.01 V, an average *J*_SC_ of 16.07 ± 0.17 mA cm^−2^, and an average FF of 80.71 ± 0.85%. Furthermore, we assessed the long‐term stability of the AsnCl‐based all‐perovskite tandem cells in an N_2_‐filled glovebox at room temperature. Remarkably, the AsnCl‐based tandems retained ≈100% of their original PCE after 1800 h (Figure S14, Supporting Information), signaling good stability of the devices.

## 3 Conclusion

In summary, we proposed a multiple‐functional additive selection rule and developed a facile yet effective method utilizing the resulting AsnCl additive to suppress Sn^2+^ oxidation, reduce Sn vacancy defects, and enhance the photovoltaic performance of mixed Sn‐Pb perovskites. This strategy effectively limited non‐radiative recombination, leading to substantial improvements in the photovoltaic parameters of mixed Sn‐Pb PSCs. The best‐performing AsnCl‐treated NBG PSC exhibited a PCE of 22.54% via a reverse *J*–*V* scan, with a comparable steady‐state PCE of 22.54%. Additionally, both long‐term storage stability and operational stability were markedly enhanced due to reduced Sn^2+^ oxidation and decreased defect density, enabling the AsnCl‐modified PSCs to retain over 90% of their initial PCE after 2500 h of storage in the dark at room temperature and ≈ 80% of their initial PCE after 210 h of constant MPP tracking at ≈55 °C. By integrating optimized NBG perovskite subcells into all‐perovskite tandem solar cells, we attained a steady‐state PCE of 28.24%. This approach demonstrates a promising pathway for developing high‐quality mixed Sn‐Pb perovskites, paving the way for efficient NBG PSCs and all‐perovskite tandem solar cells and delivering a framework for targeted molecular engineering in perovskite photovoltaics.

## Conflict of Interest

The authors declare no conflict of interest.

## Supporting information



Supporting Information

## Data Availability

The data that support the findings of this study are available from the corresponding author upon reasonable request.
