# Supplementary material for: A Multifunctional Polar Amino Acid for Mixed Tin‐Lead Perovskites and All‐Perovskite Tandems
Source: Adv Sci (Weinh). 2025 Nov 18;13(6):e10740. doi: 10.1002/advs.202510740 (PMC12866881; doi:10.1002/advs.202510740)
Supplement: Supplementary file 1 — Supporting Information [file ADVS-13-e10740-s001.docx]

Supporting Information

**A multifunctional polar amino acid for mixed tin-lead perovskites and all-perovskite tandems**

*Jin Zhou, Hongsen Cui, Chen Wang, Dexin Pu, Lishuai Huang, Shun Zhou, Guang Li, Qingxian Lin, Shining Zhang, Weiqing Chen, Guojia Fang,** *Weijun Ke,** *and Weiwei Meng**

J. Zhou

College of Physics

Hebei Advanced Thin Films Laboratory

Hebei Normal University

Shijiazhuang 050024, China

J. Zhou, H. Cui, C. Wang, D. Pu, L. Huang, S. Zhou, G. Li, Q. Lin, S. Zhang, W. Chen, G. Fang, W. Ke

Key Laboratory of Artificial Micro- and Nano-structures of Ministry of Education of China

School of Physics and Technology

Wuhan University

Wuhan 430072, China

Email: [gjfang@whu.edu.cn](mailto:gjfang@whu.edu.cn); [weijun.ke@whu.edu.cn](mailto:weijun.ke@whu.edu.cn)

W. Meng

College of Engineering

Huazhong Agricultural University

Wuhan 430070, China

E-mail: [wwmeng@m.scnu.edu.cn](mailto:wwmeng@m.scnu.edu.cn)

**Experimental Section**

**Materials**

All materials were used as received without further purification. Methylammonium iodide (MAI), formamidinium iodide (FAI), lead iodide (PbI_2_), tin (II) iodide (SnI_2_), and lead bromide (PbBr_2_) were purchased from Advanced Election Technology Co., Ltd. PEDOT: PSS aqueous solutions, bathocuproine (BCP), and fullerene (C_60_) were purchased from Xi’an Polymer Light Technology. Ethane-1,2-diammonium iodide (EDAI_2_), tin(II) fluoride (SnF_2_), cesium iodide (CsI), isopropanol (IPA), N,N-dimethylformamide (DMF), dimethyl sulfoxide (DMSO), and chlorobenzene were purchased from Sigma-Aldrich. [4-(3,6-Dimethyl-9H-carbazol-9-yl)butyl]phosphonic acid (Me-4PACz) was purchased from TCI. Asparaginate was purchased from Bidepharm. Patterned ITO substrates (12 Ω per square) with dimensions of 20 × 20 mm were purchased from Advanced Election Technology Co., Ltd.

**Narrow-bandgap (NBG) FA_0.7_MA_0.3_Pb_0.5_Sn_0.5_I_3_ perovskite precursors and post-treatment solutions**

Firstly, the precursor solutions (2.2 M) were prepared by dissolving FAI, MAI, PbI_2_, and SnI_2_ in a mixed solvent of DMF and DMSO at a volume ratio of 3:1. SnF_2_ (5 mol% with respect to the B-site) Pb(SCN)_2_ (1 mol% with respect to the A-site) were also added to the precursor solutions. The solutions were then stirred for 1 h in an N_2_-filled glovebox. Finally, the solutions were filtered through 0.22 μm polytetrafluoroethylene (PTFE) membranes before use.

The post-treatment solutions were prepared by dissolving EDAI_2_ in IPA at a concentration of 0.5 mg ml^−1^. To accelerate the dissolving process of EDAI_2_, the solutions were heated at 40 ℃ for ~4 h in an N_2_-filled glovebox before use. The EDAI_2_ post-treatment is inspired by the former study of Wakamiya et al.^[1]^

**Wide-bandgap (WBG) FA_0.8_Cs_0.2_Pb(I_0.6_Br_0.4_)_3_ perovskite precursor solutions**

The precursor solutions (1.2 M) were prepared by dissolving FAI, CsI, PbBr_2_, and PbI_2_ in a mixed solvent of DMF and DMSO at a volume ratio of 4:1. The solutions were stirred for 1 h at 60℃ and then filtered through 0.22 μm poly(vinylidene fluoride) membranes before use.

**NBG perovskite solar cell fabrication**

ITO substrates were cleaned in acetone and ethanol for 15 min each. UVO treatment was subsequently conducted for further cleaning. PEDOT:PSS solutions were spin-coated onto the ITO substrates at a speed of 5000 rpm for 30 s, followed by annealing at 140℃ for 20 min in ambient air. After cooling, the ITO substrates were transferred to an N_2_-filled glovebox. Perovskite films were fabricated via a two-step spin-coating process: (1) 1000 rpm for 10 s with an acceleration of 200 rpm s^−1^ and (2) 4000 rpm for 40 s with an acceleration of 1000 rpm s^−1^. 400 μL of chlorobenzene was dropped onto the spinning ITO substrates at the 20^th^ s before the end of the total spin-coating process. The ITO substrates were subsequently annealed at 100 ℃ for 10 min. Post-treatments were conducted by spin-coating the solutions of EDAI_2_ on the perovskite films at 4000 rpm for 30 s, followed by annealing at 100 ℃ for 5 min. At last, C_60_ (20 nm), BCP (7 nm), and Cu (100 nm) layers were sequentially deposited on the perovskite films via thermal evaporation.

**All-perovskite tandem fabrication**

ITO substrates were cleaned as described above. NiO_x_ layers were prepared by spin-coating a NiO_x_ solution (10 mg ml^−1^ in pure water) at 3000 rpm for 30 s and heating at 130℃ for 30 min. Me-4PACz (0.3 mg ml^−1^ in ethanol) layers were spin-coated onto the ITO substrates at 3000 rpm for 30 s, followed by heating at 100 ℃ for 10 min in an N_2_-filled glovebox. For WBG perovskite fabrication, 50 μL of precursor solutions were dropped onto each substrate and spin-coated at 5000 rpm for 60 s, with 300 μL of diethyl ether dropped 30 s before the end. The substrates were annealed at 50℃ for 2 min and 100℃ for 10 min. Afterward, 1,3-propane-diammonium iodide (PDAI_2_) post-treatment was conducted by spin-coating PDAI_2_ solutions (in IPA, 2 mg ml^−1^) at 4000 rpm for 30 s, followed by an annealing process at 100 °C for 5 min. The C_60_ film (a thickness of 18 nm at a rate of 0.005 nm/s) was deposited onto the WBG perovskites via thermal evaporation. Then, the ALD SnO_x_ layer (20 nm) and Au layer (a thickness of 0.8 nm at a rate of 0.005 nm/s) were deposited via thermal evaporation. Pristine PEDOT:PSS was diluted with ethanol (1:1 volume ratio) and spin-coated onto the Au layer at 5000 rpm for 30 s, followed by heating at 100 ℃ for 10 min. The substrates were then transferred to an N_2_-filled glovebox for NBG film fabrication, as described above. Finally, C_60_ (a thickness of 20 nm at a rate of 0.005 nm/s), BCP (a thickness of 7 nm at a rate of 0.005 nm/s), and Cu (a thickness of 100 nm at a rate of 0.05 nm/s) were deposited in sequence.

**Film and device characterization**

*J-V* characteristics of the devices were measured using a Keithley 2400 source meter under AM 1.5G illumination in an N_2_-filled glovebox at ~26℃. The light intensity was set to 100 mW cm^-2^ and it was calibrated by a certified WPVS standard solar reference cell (SRC-2020, Enlitech; traceable to NREL) before the measurements. For the *J-V* scans of NBG cells, the scan rate was 0.02 V s^–1^, the voltage step was 50 mV, and the delay time was 25 ms. Forward scans were conducted from -0.1 V to 1 V, and reverse scans were conducted from 1 V to -0.1 V. The cell area was 0.0948 cm^2^, and an aperture shade mask with an area of 0.070225 cm^2^ was used during the *J-V* tests. EQE curves were measured using an EQE system (Enli Technology Co., Ltd) by focusing monochromatic light onto device pixels in ambient air, calibrated using a standard silicon photodiode.

X-ray diffraction measurements and pole figure characterizations of perovskite crystals were performed using an X-ray diffractometer (Bruker AXS, D8 Advance). Scanning electron microscopy images of films and cells were obtained using a Zeiss SIGMA field-emission scanning electron microscope. The UV-vis absorption spectra of films were measured using a SHIMADZU mini 1280 UV-vis spectrophotometer. Electronic impedance spectroscopy spectra were obtained using a CHI 770E electrochemical workstation (Shanghai Chenhua Instruments, China) in the frequency range of 1 MHz to 1 Hz. X-ray photoelectron spectroscopy tests were performed using a photoelectron spectrometer (Thermo Scientific, ESCLAB 250Xi, USA). Morphology images were captured with a Zeiss microscope. Light absorption spectra were recorded using a SHIMADZU mini 1280 UV-visible spectrophotometer.

**Density functional theory (DFT) calculations**

The DFT calculations are carried out using the Vienna ab initio simulation package.^[2, 3]^ The core−valence interaction is described by the projector-augmented wave method.^[4, 5]^ The cutoff energy for basis functions was 520 eV. The generalized gradient approximation (GGA) of the Perdew−Burke−Ernzerh (PBE) functional was used for exchange correlation.^[6]^ The Grimme’s DFT-D3 scheme was employed for the molecular system.^[7]^ All atoms were relaxed until the Hellmann−Feynman forces on them were below 0.03 eV Å^‒1^. Single-molecule model within a vacuum box (25Å along three directions) and G-only k point mesh were chosen. The visualization of crystal structures was done using VESTA software.^[8]^ The maximum and minimum 𝜑 values of the functional groups were obtained with the help of the Multiwfn code.^[9]^

**
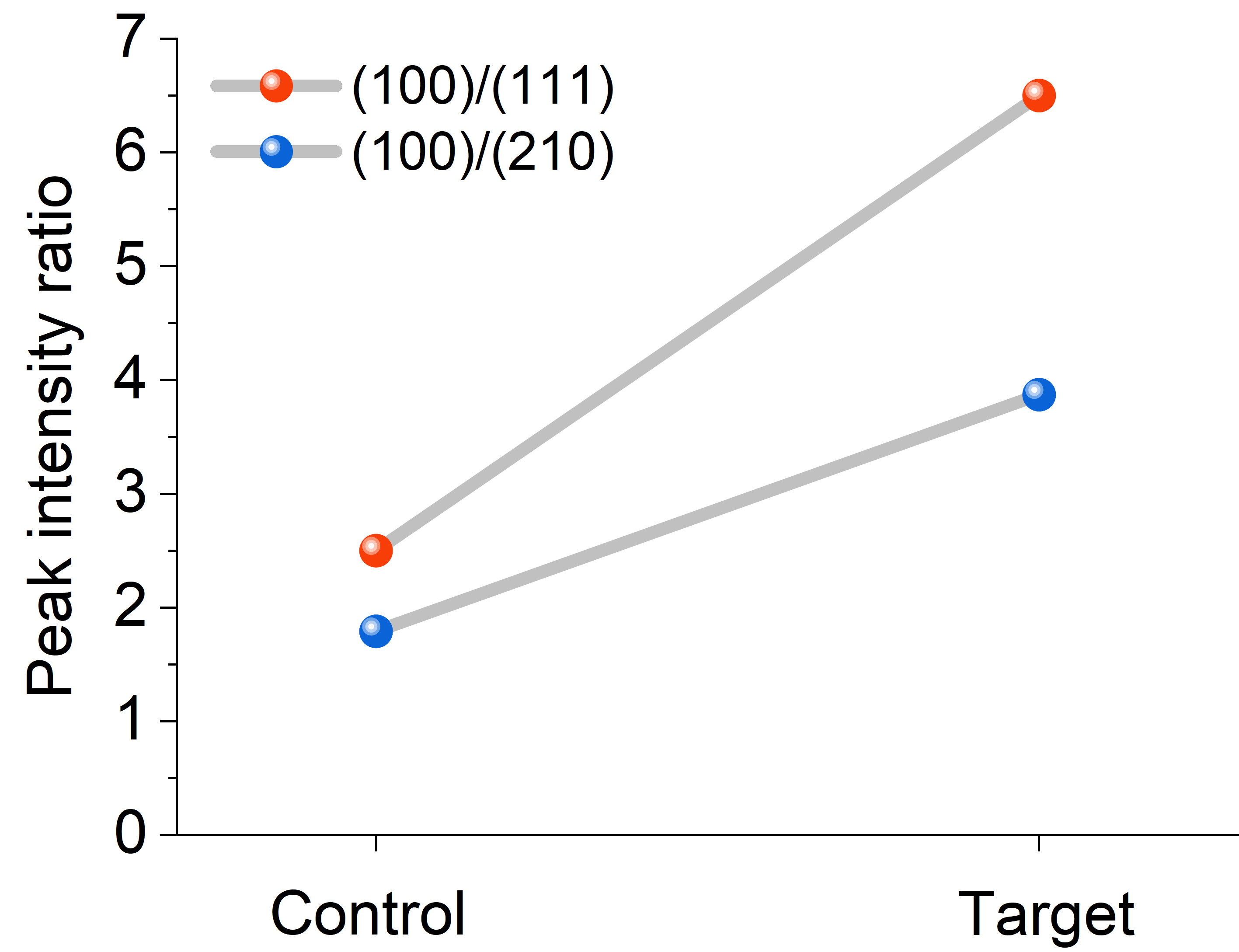
**

**Figure S1.** Intensity ratios between various perovskite lattice planes extracted from XRD patterns.


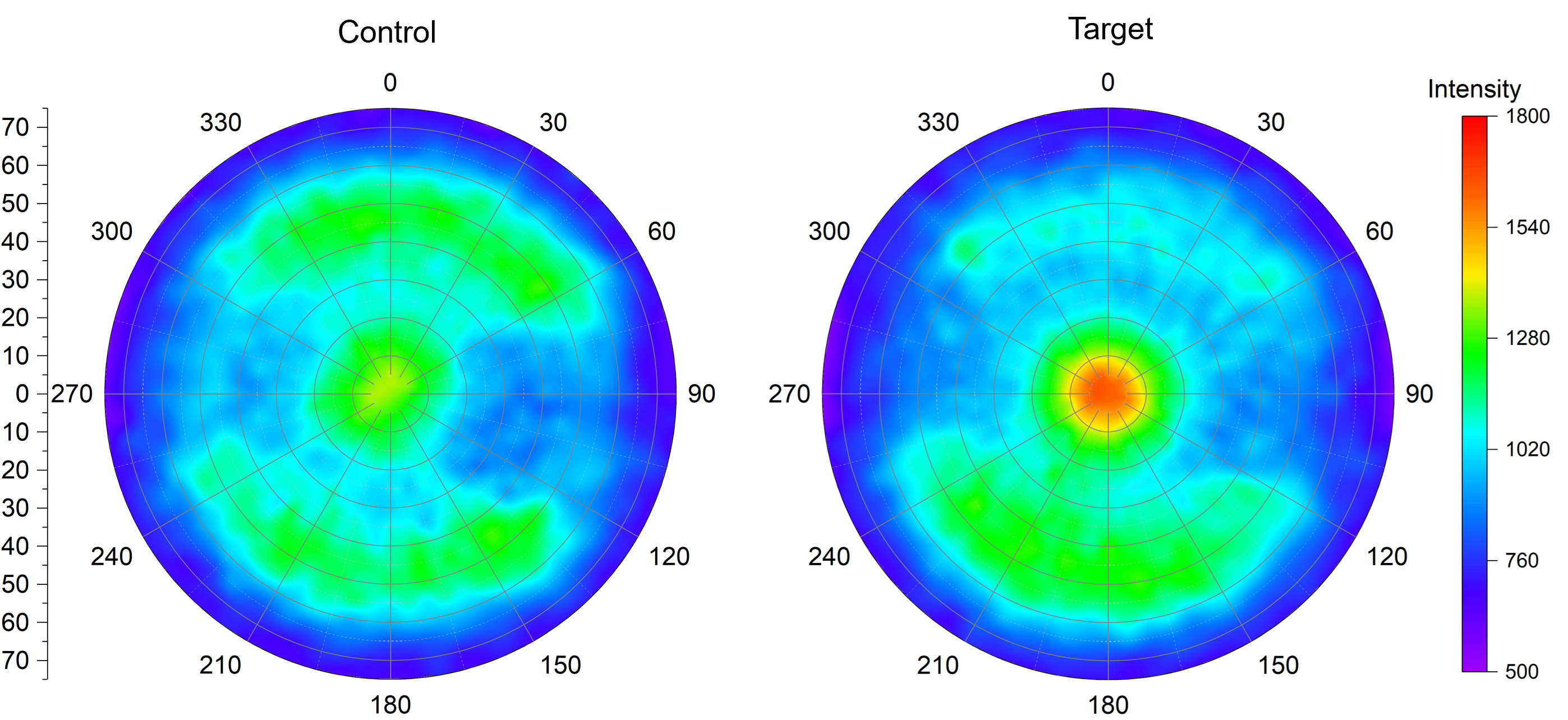


**Figure S2.** Pole figure measurements of the (100) facet orientation.

**
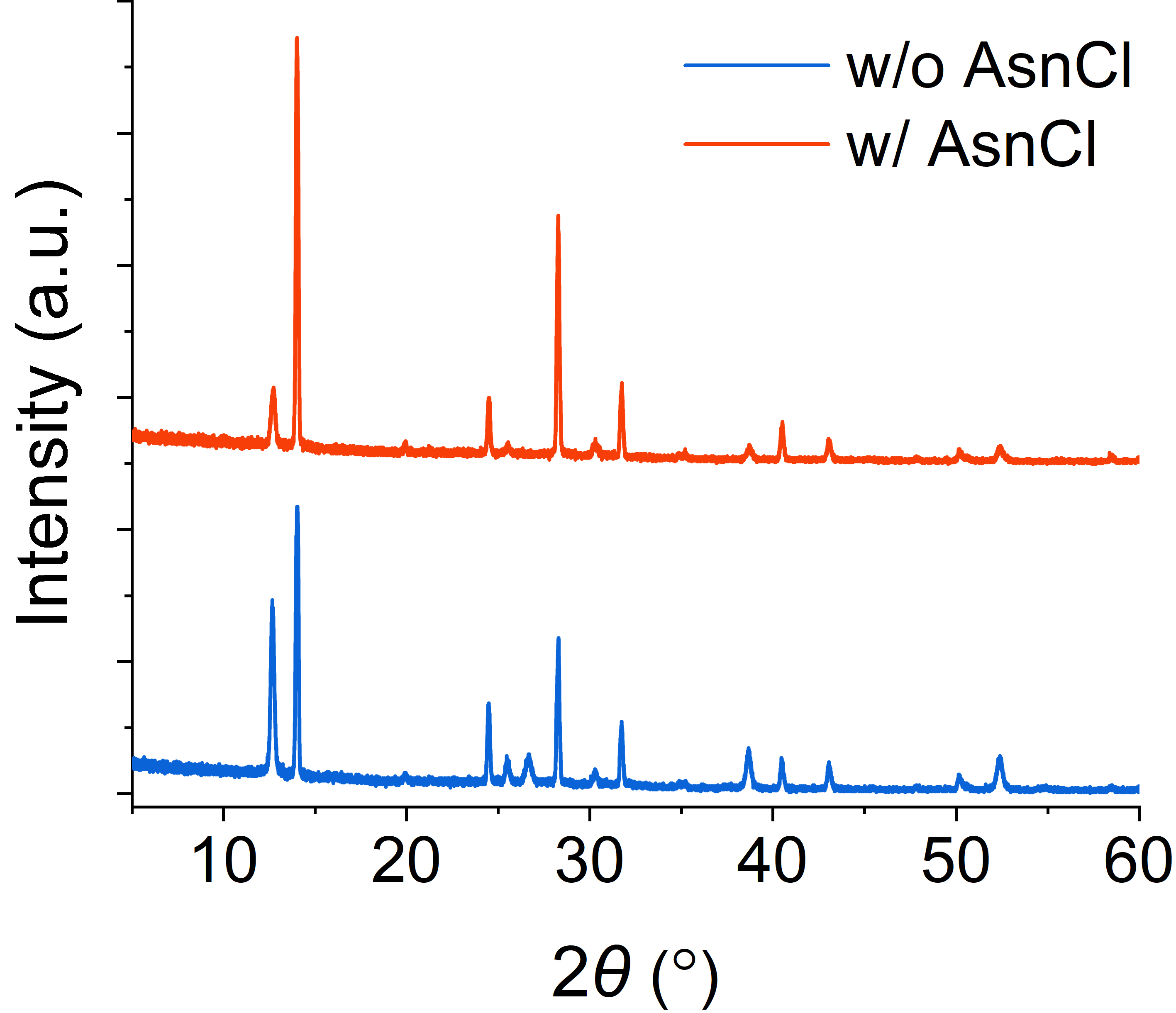
**

**Figure S3.** XRD patterns of mixed Sn-Pb perovskites with 5% excess PbI_2_, without and with AsnCl incorporation.

**
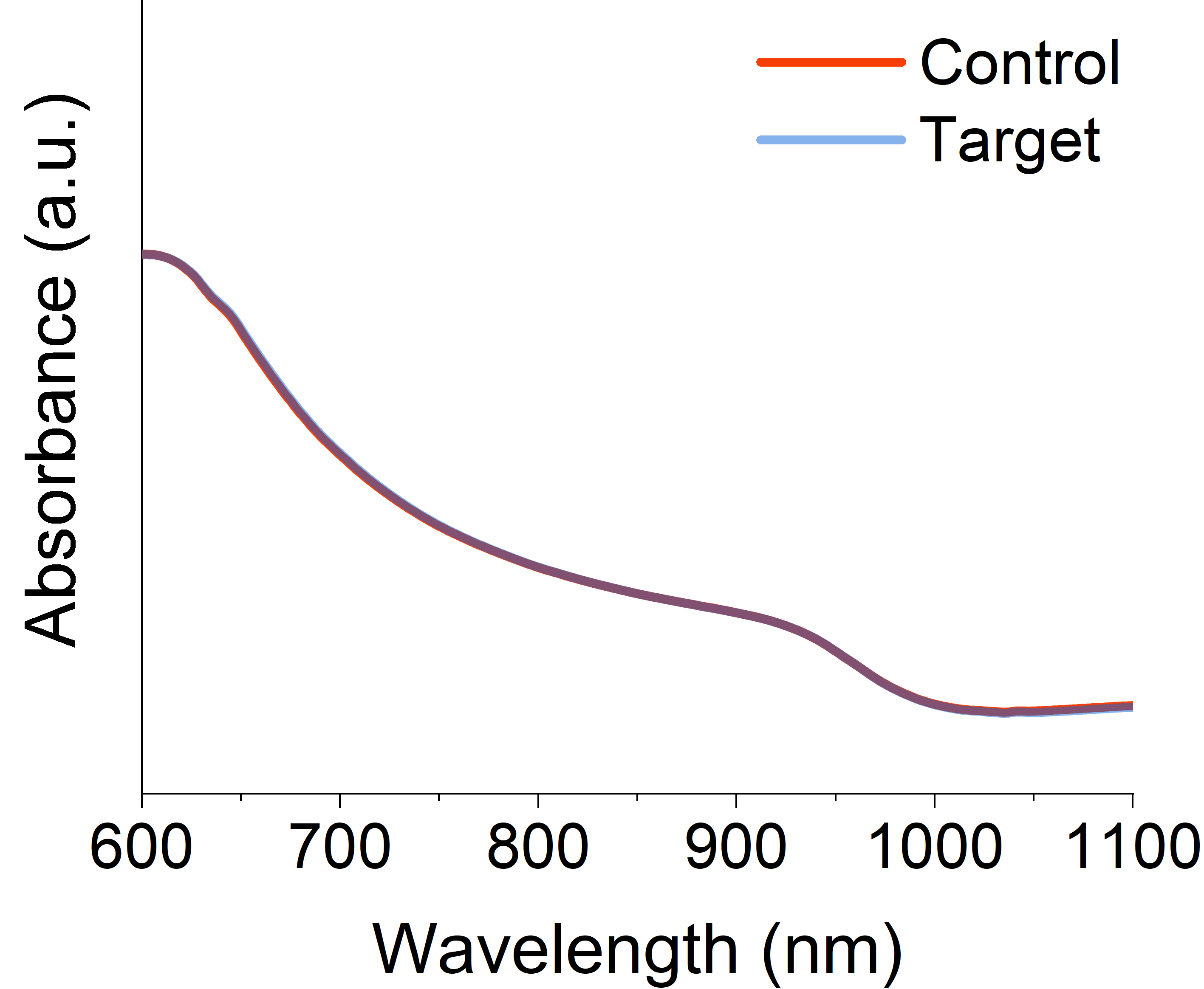
**

**Figure S4.** Ultraviolet-visible absorption of control and AsnCl-modified mixed Sn-Pb perovskite films.


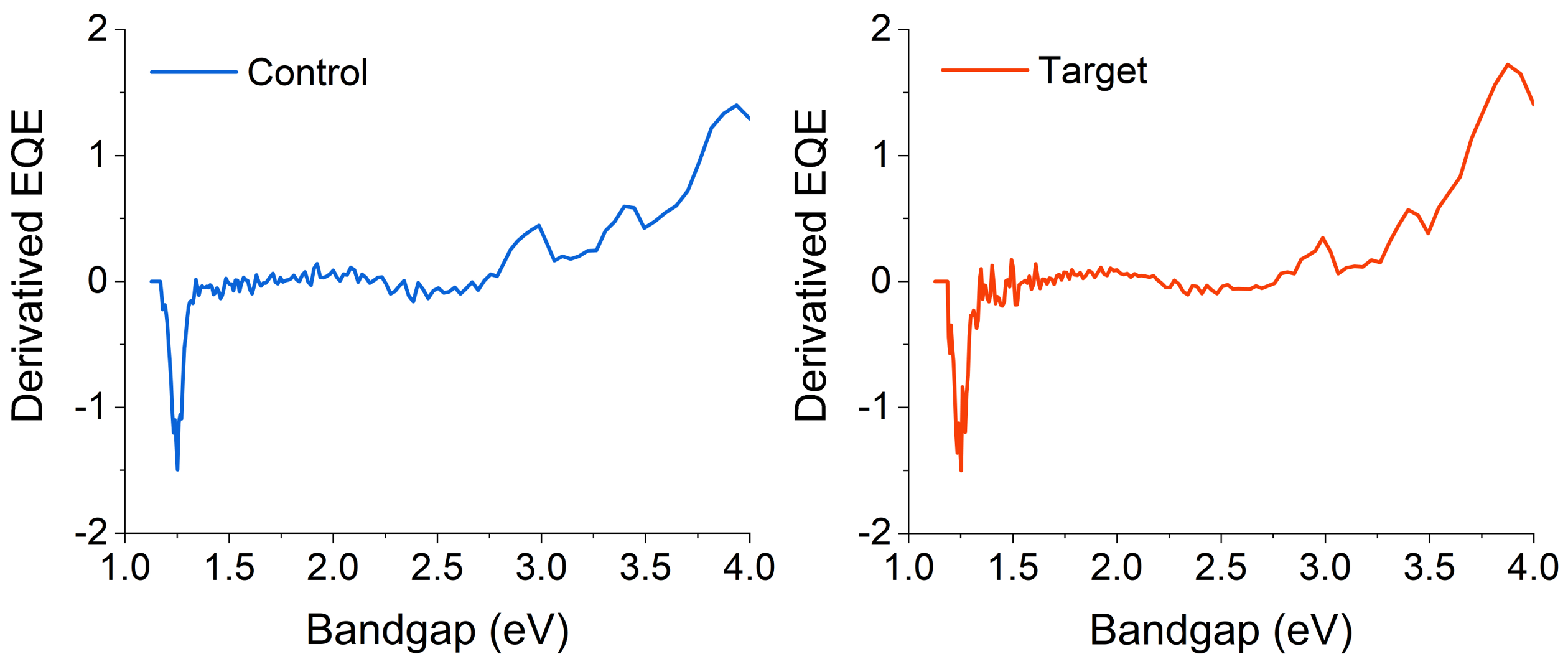


**Figure S5.** The bandgaps of control and target mixed Sn-Pb perovskites determined by Gaussian distribution of dEQE/dE.

**
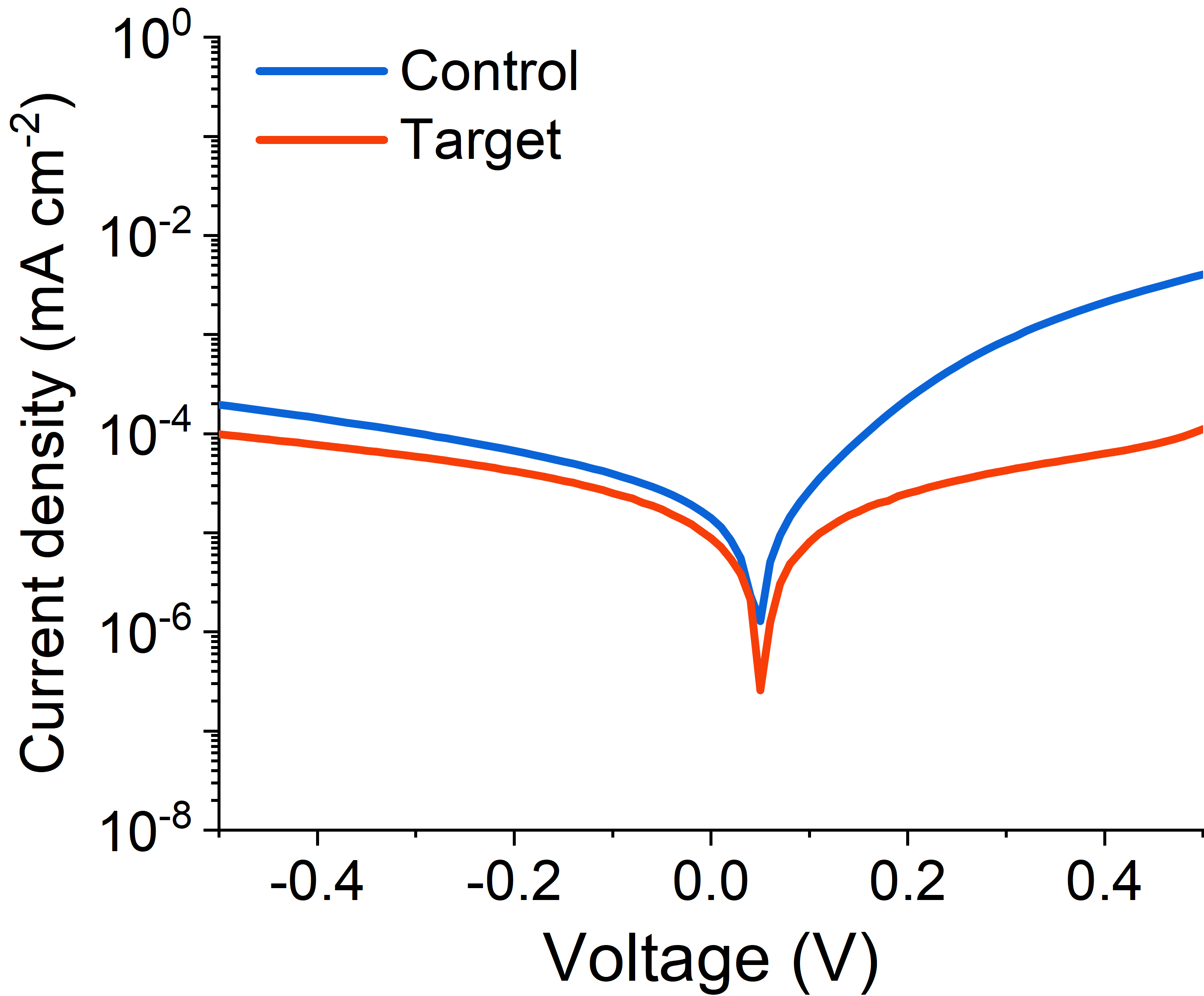
**

**Figure S6**. Dark *J*-*V* curves of control and AsnCl-modified mixed Sn-Pb PSCs.


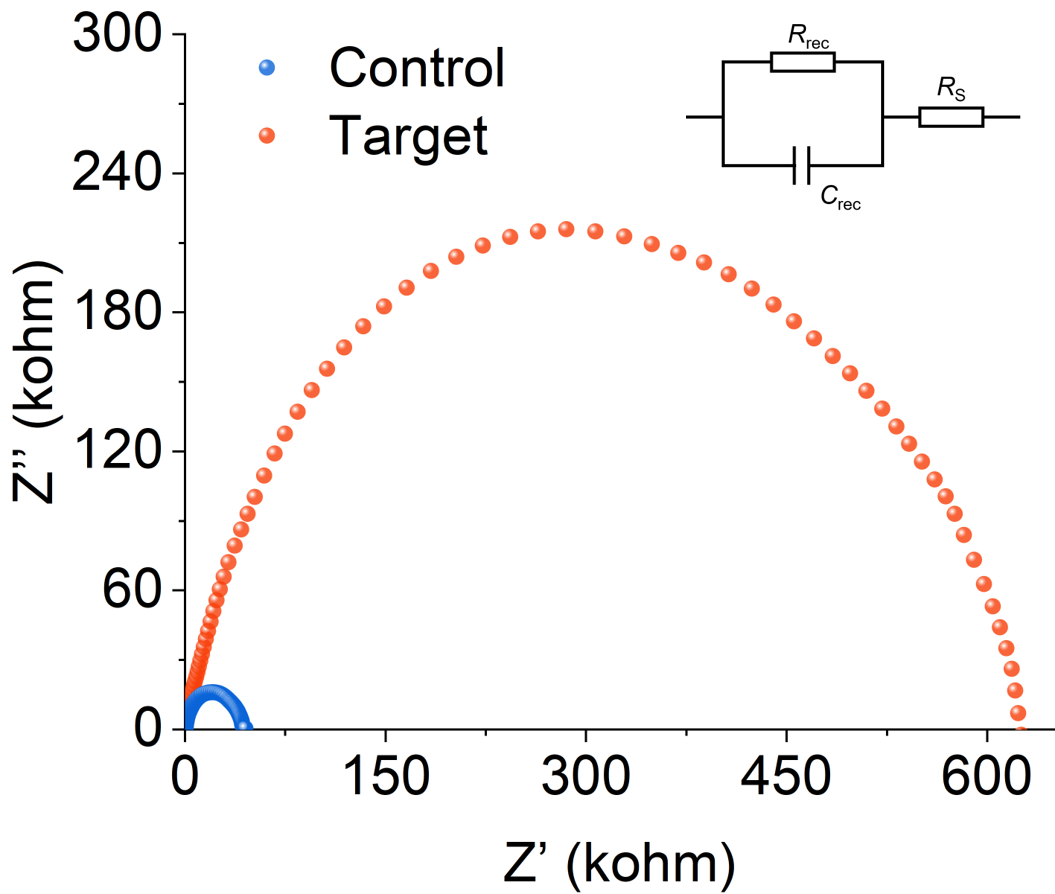


**Figure S7**. Nyquist plots of control devices and AsnCl-incorporated devices. Inset: equivalent circuit.


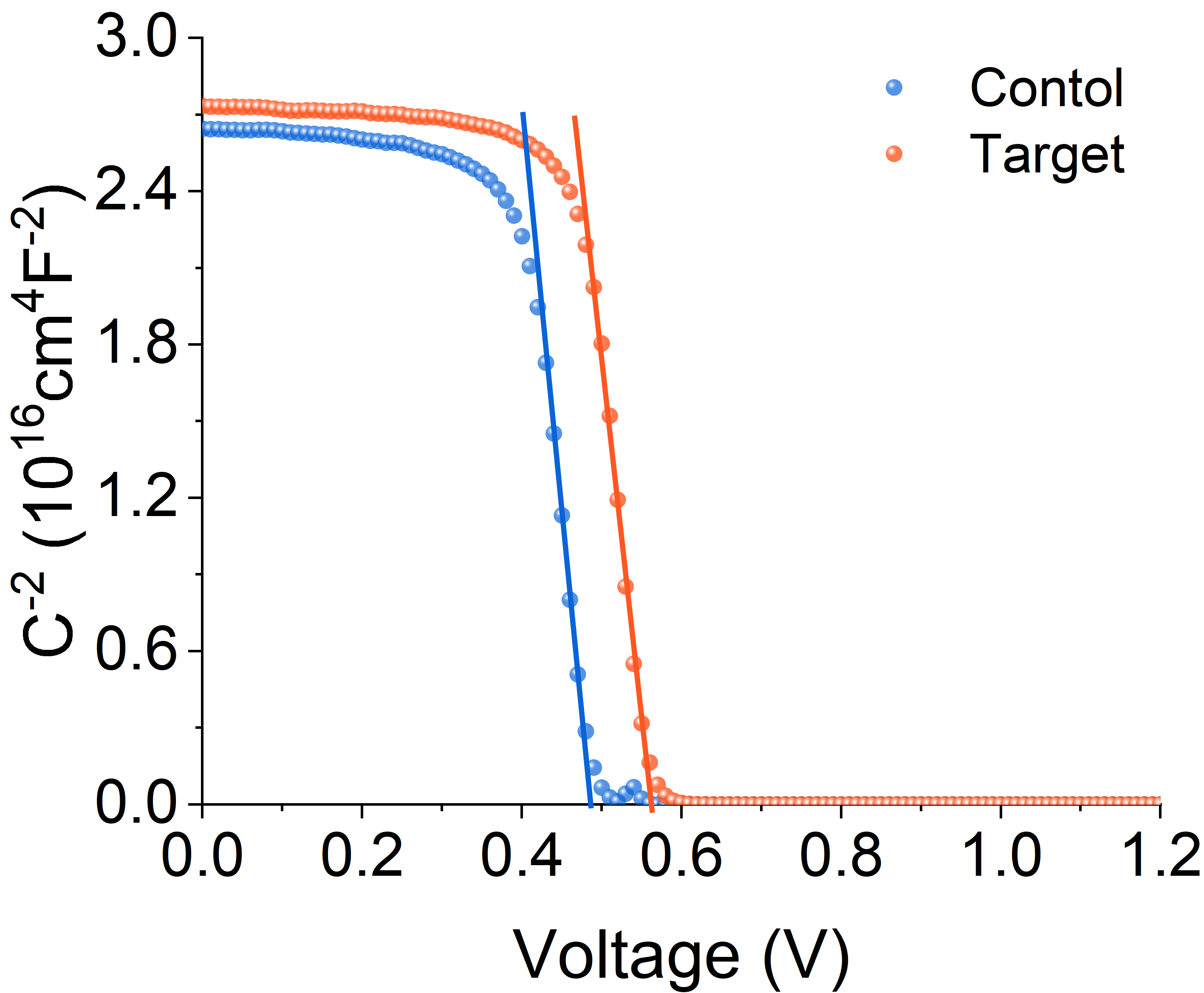


**Figure S8**. Mott-Schottky curves of control and AsnCl-incorporated PSCs.


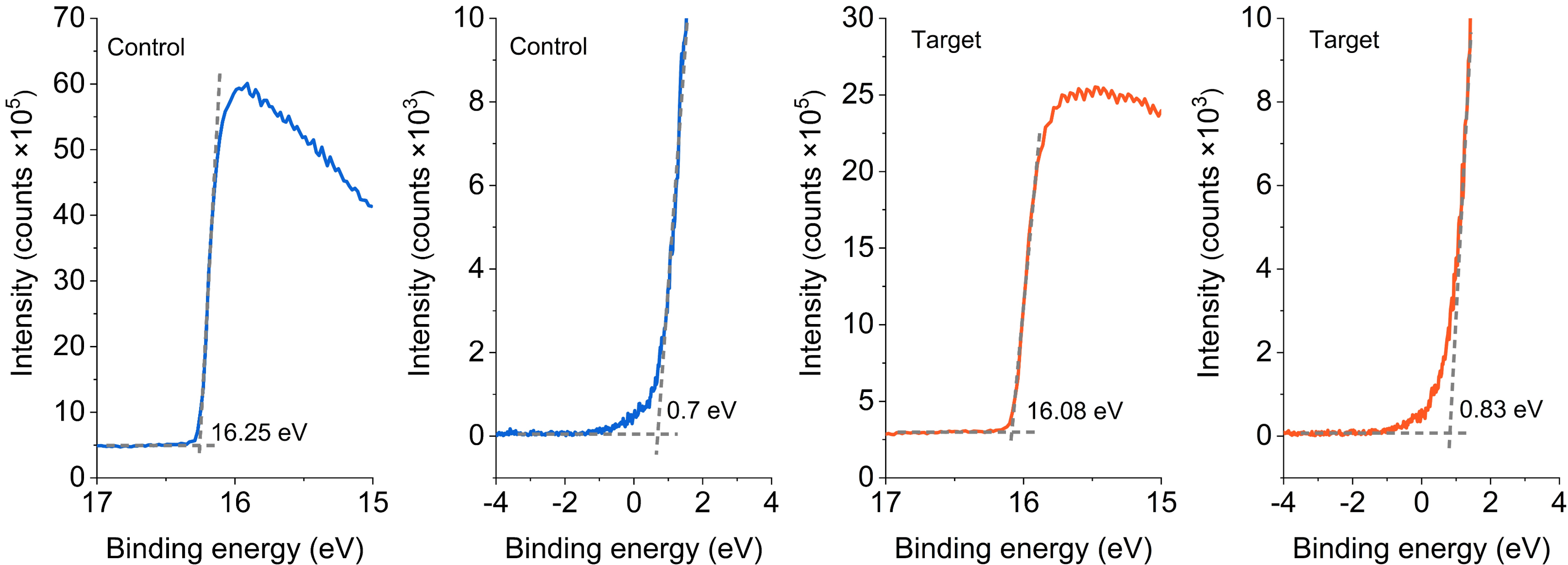
 **Figure S9**. UPS measurements of perovskite films with and without AsnCl treatment.


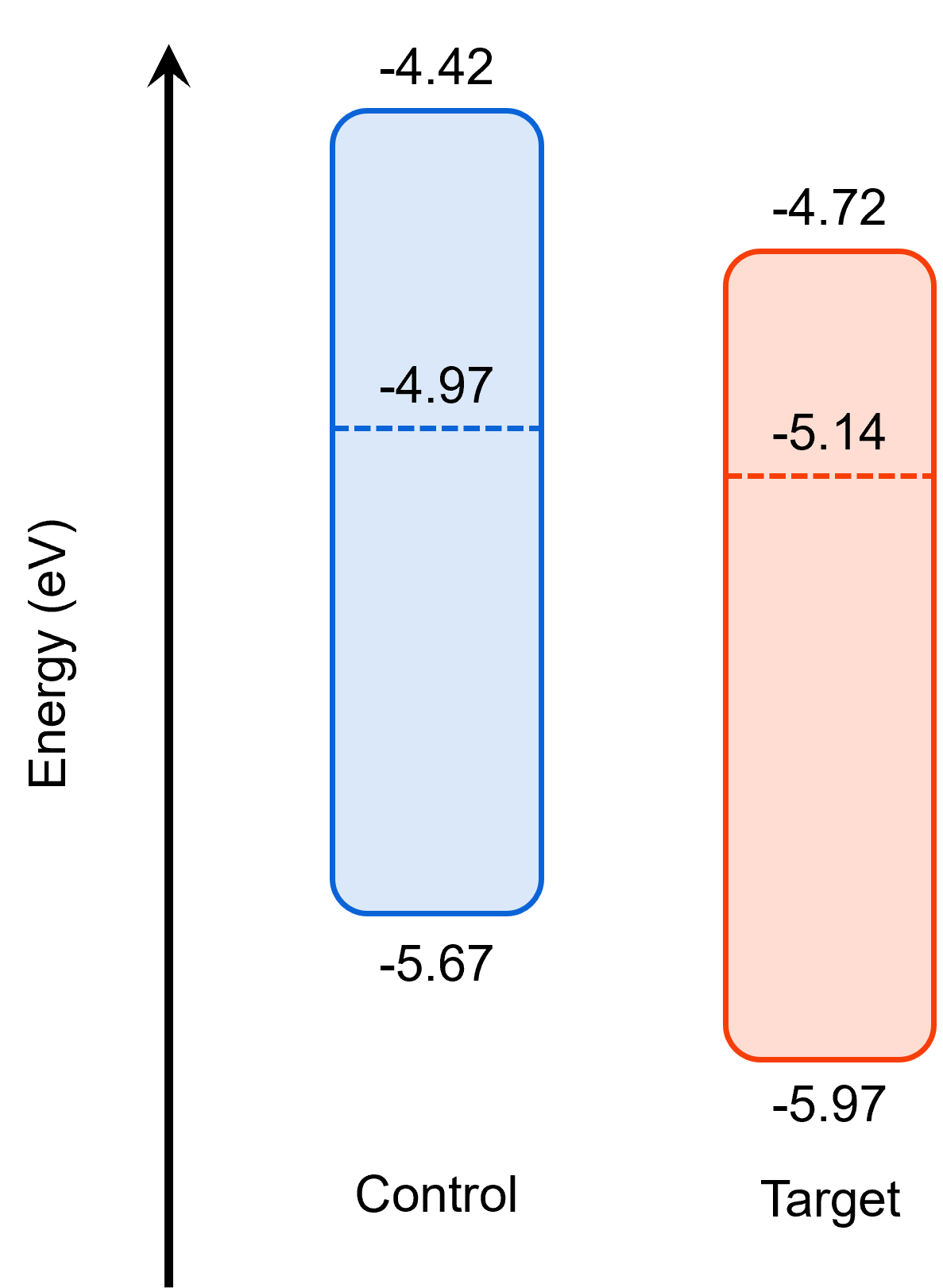


**Figure S10**. Calculated CBM, VBM, and Fermi levels of control and AsnCl-incorporated perovskites.


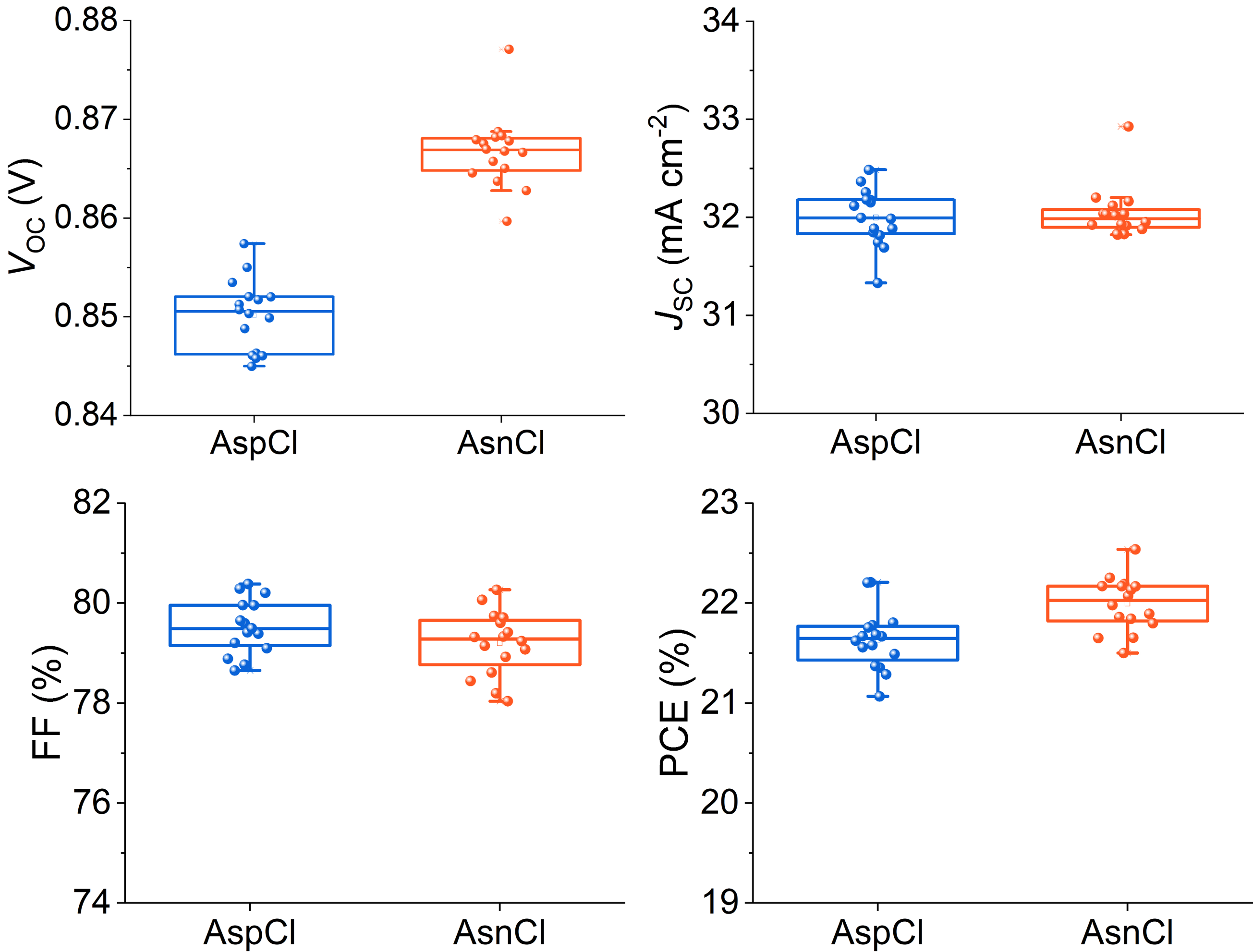


**Figure S11**. Photovoltaic parameters of Asp-treated and AsnCl-modified mixed Sn-Pb PSCs.

**
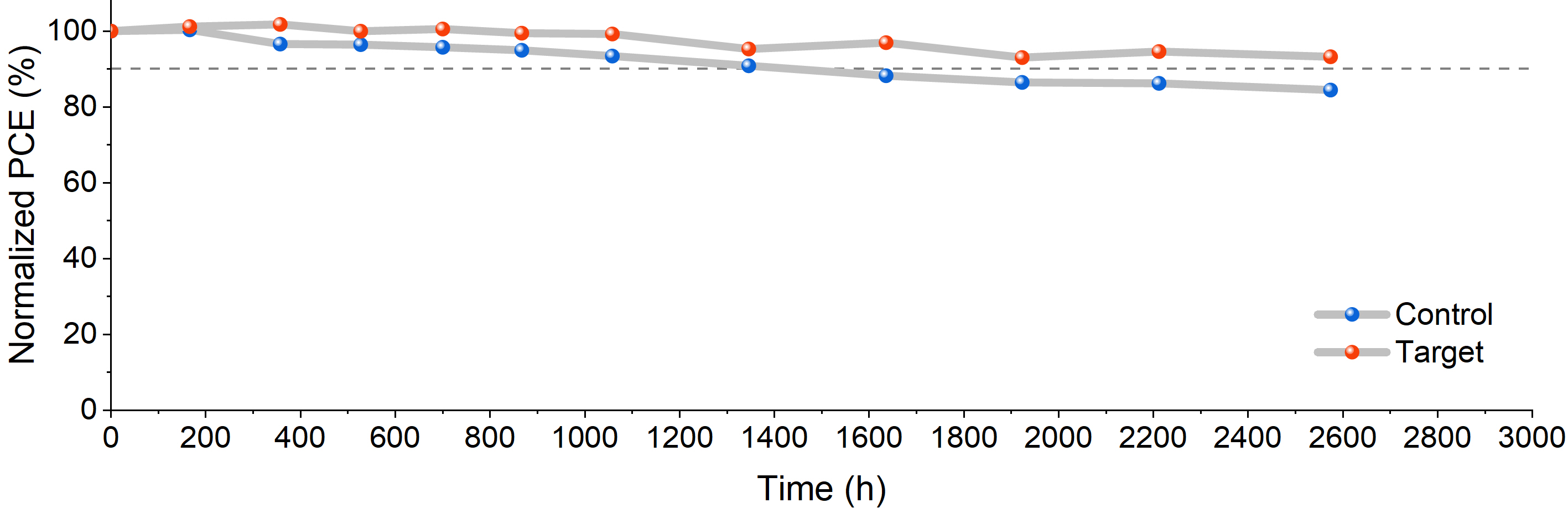
 Figure S12**. Long-term stability of single-junction NBG PSCs.


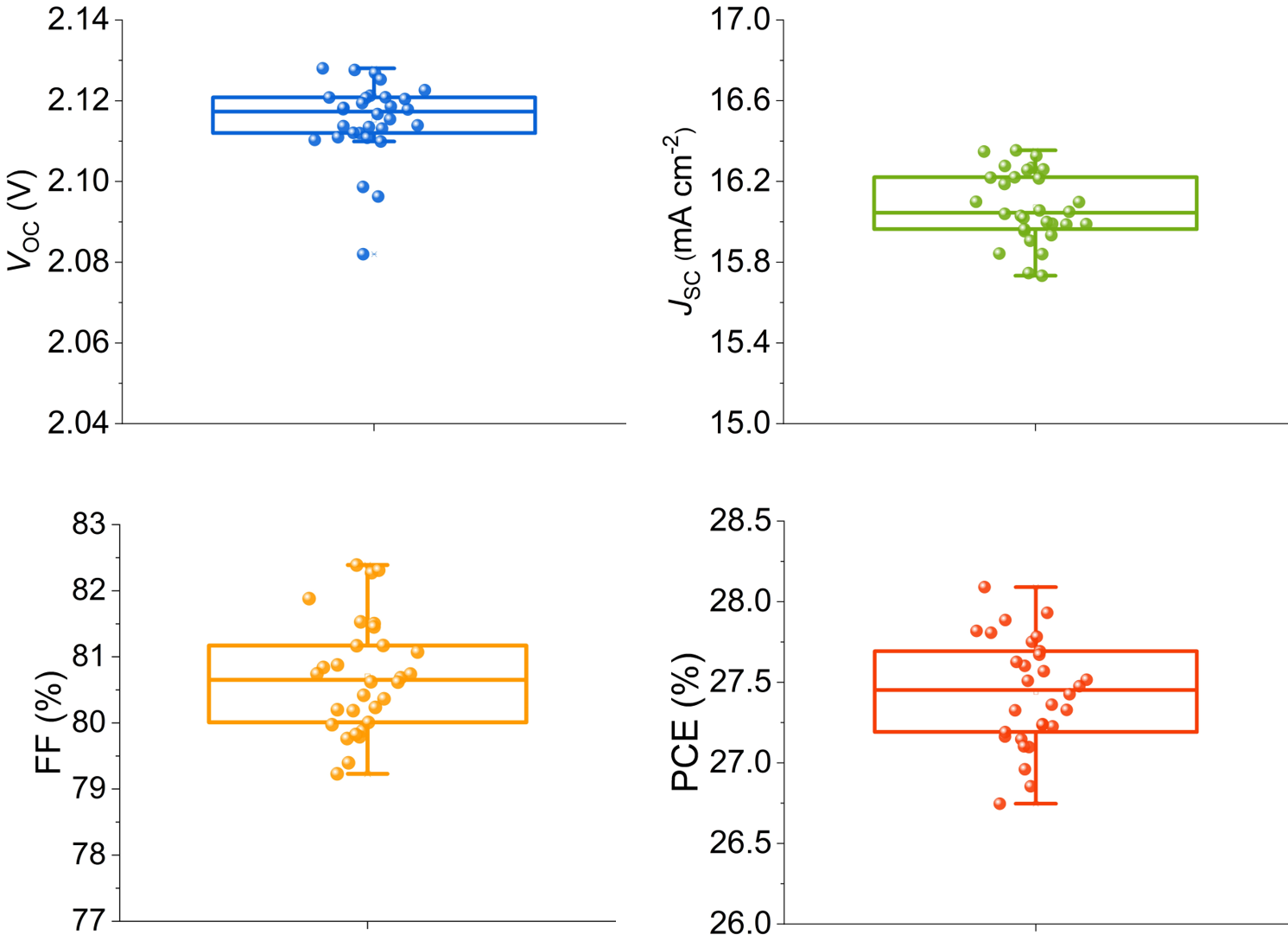


**Figure S13**. Photovoltaic parameters of AsnCl-modified all-perovskite tandem PSCs.

**
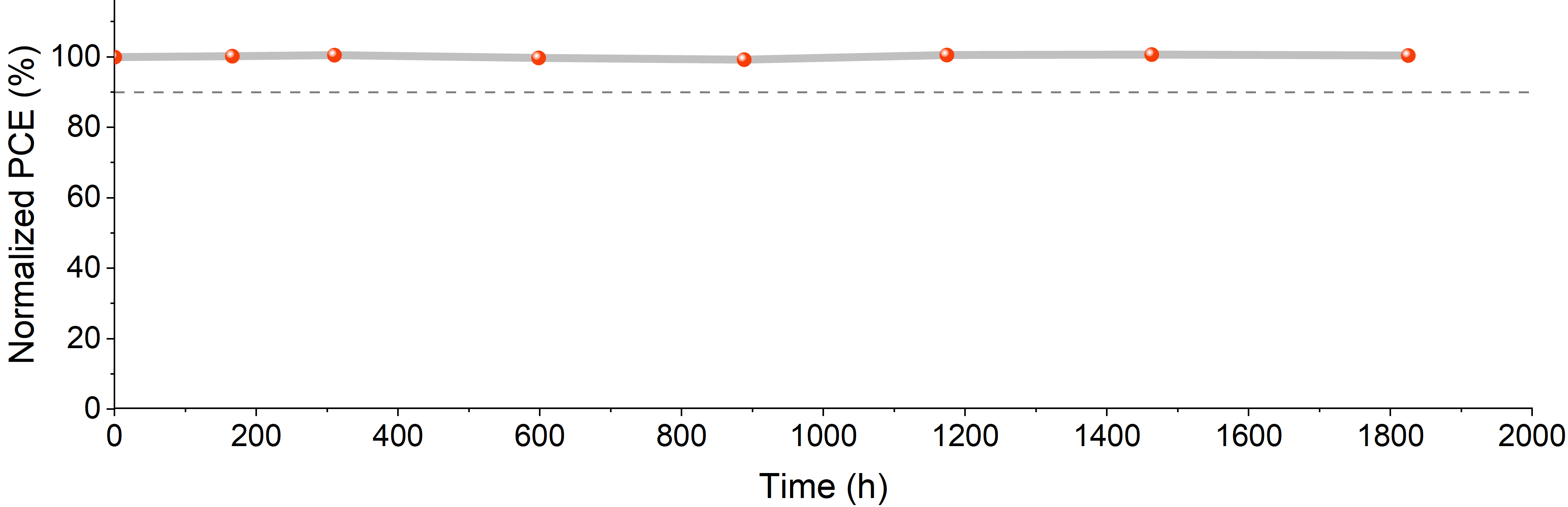
 Figure S14**. Long-term stability of an AsnCl-based all-perovskite tandem solar cell.

**References**

[1] S. Hu, et al., *Energy Environ. Sci.* **2022**, *15*, 2096.

[2] G. Kresse, J. Hafner, *Phys. Rev. B* **1993**, *47*, 558.

[3] G. Kresse, J. Furthmuller, *Phys. Rev. B* **1996**, *54*, 11169.

[4] P. E. Blochl, *Phys. Rev. B* **1994**, *50*, 17953.

[5] G. Kresse, D. Joubert, *Phys. Rev. B* **1999**, *59*, 1758.

[6] J. P. Perdew, et al., *Phys. Rev. Lett.* **1996**, *77*, 3865.

[7] S. Grimme, et al., *J. Chem. Phys.* **2010**, *132*, 154104.

[8] K. Momma, F. Izumi, *J. Appl. Cryst.* **2011**, *44*, 1272.

[9] T. Lu, F. Chen, *J. Comput. Chem.* **2012**, *33*, 580.
